# Daily Ingestion of Eggplant Powder Improves Blood Pressure and Psychological State in Stressed Individuals: A Randomized Placebo-Controlled Study

**DOI:** 10.3390/nu11112797

**Published:** 2019-11-16

**Authors:** Mie Nishimura, Miho Suzuki, Ryuto Takahashi, Shohei Yamaguchi, Kazufumi Tsubaki, Tomoyuki Fujita, Jun Nishihira, Kozo Nakamura

**Affiliations:** 1 Department of Medical Management and Informatics, Hokkaido Information University, Hokkaido 069-8585, Japan; mnishimura@do-johodai.ac.jp; 2 Department of Agriculture, Graduate School of Science and Technology, Shinshu University, Nagano 399-4598, Japan; 19as208c@shinshu-u.ac.jp (M.S.); 18as208k@shinshu-u.ac.jp (R.T.); tfujita@shinshu-u.ac.jp (T.F.); 3 Department of Science and Technology, Graduate School of Medicine, Science and Technology, Shinshu University, Nagano 399-4598, Japan; 19hs505d@shinshu-u.ac.jp; 4 Future Business Search Team, Planning Department, R & D Division, ADEKA co., Tokyo 116-8554, Japan; tsubaki-ka@adeka.co.jp; 5 Institute of Agriculture, Academic Assembly, Shinshu University, Nagano 399-4598, Japan

**Keywords:** acetylcholine, eggplant, blood pressure, psychological state, randomized controlled study

## Abstract

Eggplant (*Solanum melongena*) is a globally popular vegetable and its significant health effect has not been reported in randomized controlled trials. Recently, we reported that eggplant was rich in choline esters, including acetylcholine (ACh), and had an antihypertensive effect in spontaneously hypertensive rats. Here, we evaluated the effects of a continuous intake of eggplant powder on blood pressure (BP), stress, and psychological state (PS) in 100 stressed participants with normal-high BP or grade 1 hypertension in a randomized, double-blind, placebo-controlled, parallel-group comparative study. The participants were randomly assigned to the eggplant or placebo group. Participants in the eggplant group ingested capsules containing eggplant powder (1.2 g/day; 2.3 mg of ACh/day) for 12 weeks, whereas participants in the placebo group ingested placebo capsules. The primary outcome assessed was hospital BP. Secondary outcomes were stress and PS. Eggplant powder intake significantly decreased the hospital diastolic blood pressure (DBP) at week 8 overall and in the normal-high BP group, and the systolic blood pressure (SBP) and DBP at week 12 overall and in the grade 1 hypertension group, compared to those of the placebo group. It also improved negative PSs at week 8 or 12 in the normal-high BP group. This is the first evidence of the BP- and PS-improving effects of eggplant intake in humans. The functional substance responsible for the effects was estimated to be eggplant-derived choline ester, namely ACh.

## 1. Introduction

According to a report published on World Health Day, cardiovascular disease accounts for approximately 17 million deaths per year, corresponding to almost one third of total global mortality [1]. Hypertension accounts for 9.4 million deaths worldwide every year, contributing to at least 45% of deaths attributed to heart disease and 51% of those attributed to stroke. Hypertension also contributes to kidney failure, premature mortality, and disability [1]. Therefore, prevention and management of hypertension are major public health challenges worldwide. Several studies have shown that lifestyle modifications, including dietary changes, physical exercise, and weight loss, can contribute to the primary prevention of hypertension and significantly reduce blood pressure (BP) in people with established hypertension [2,3,4]. Functional foods with antihypertensive effects are also expected to prevent and manage hypertension [5]. In Japan, functional foods for people with higher than normal BP are commercially available as government-approved “Food for Specified Health Uses” or “Foods with Function Claims” with the food business operator’s own responsibility.

Eggplant (*Solanum melongena*) is a globally popular vegetable consumed daily, and an agronomically and economically important cultivated crop. Eggplant is low in calories, carbohydrate, and protein, and rich in dietary fiber and minerals [6]; thus, it is recommended for reducing caloric intake and body weight (BW) to prevent type-2 diabetes [7,8,9]. Our research found only two clinical trials investigating eggplant related to the cholesterol-lowering effects in hypercholesterolemic participants [10] and its efficacy in reducing fat mass in overweight women [11].

Acetylcholine (ACh) is well-known as a neurotransmitter and present in the nervous system of not only mammals but also almost all organisms, including those used as food for humans [12]. Food materials such as eggplant, bamboo shoots (*Phyllostachys bambusoides*), and shiitake mushroom (*Lentinus eddoes*) are reported to contain ACh [12,13]. We have reported the isolation of ACh and lactoylcholine from lactic acid bacteria-fermented food with a hypotensive effect in spontaneously hypertensive rats (SHRs) and reported that choline esters might be responsible for the BP-lowering effect [14,15]. Recently, we presented the antihypertensive effects of orally administered eggplant powder rich in ACh in SHRs [16] and suggested that ACh in eggplant suppressed sympathetic nervous activity to cause the effect. Bethanechol, an artificial cholinergic agent, stimulates the parasympathetic nervous system through muscarinic ACh receptor [17] and lowers BP. Therefore, ACh in orally administered eggplant was estimated to promote parasympathetic nervous activity and then suppress sympathetic nervous activity by reciprocal innervation. Stress and psychological state (PS) are closely related to sympathetic nervous activity [18,19]. Psychosocial factors are also related to blood pressure and may lead to hypertension [20]. Therefore, eggplant ingestion may improve stress and PSs, in addition to BP.

Thus, this study aimed to examine the long-term antihypertensive, anti-stress, and PS-improving effects of eggplant in human participants with normal-high BP (130 mmHg < systolic blood pressure (SBP) ≤ 139 mmHg and 85 mmHg < diastolic blood pressure (DBP) ≤ 89 mmHg) and/or grade 1 hypertension (140 mmHg < SBP ≤ 159 mmHg and 90 mmHg < DBP ≤ 99 mmHg) by a randomized, double-blind, placebo-controlled, parallel-group comparative study.

## 2. Materials and Methods

### 2.1. Study Population

We screened 189 volunteers on screening visit, and 100 Japanese participants were enrolled in the study. Participants with any of the following were excluded from the study: participants under a physician’s advice, treatment, or medication for hypertension, schizophrenia, depressive disorder, mania neurologic disorder, arrhythmia, or bradycardia; participants with a body mass index (BMI) of ≥30 kg/m^2^; pre- or post-menopausal women complaining of obvious physical changes; participants at risk of having allergic reactions to drugs or foods, especially those based on eggplant, secondary hypertension, serious cerebrovascular, cardiac, hepatic, renal, gastrointestinal diseases, or affected with infectious diseases requiring reports to the authorities; participants with history of major surgery relevant to the digestive system; participants with unusually high or low blood pressure, or abnormal hematological data; participants with severe anemia; participants who regularly received medicine, functional foods, or supplements that would affect BP or stress; participants with alcohol addiction or an eating disorder; participants who donated either 400 mL of whole blood within 16 weeks (women) or 12 weeks (men), 200 mL of whole blood within 4 weeks (men and women), or blood components within 2 weeks (men and women) prior to the current study; participants who were participating in other clinical studies, or participated within the last 4 weeks prior to the current study; pregnant or lactating women; women who expected to be pregnant during this study; and participants with any other medical or health reasons unfavorable in the current study. Eligible participants were randomly assigned to either the eggplant (capsules containing eggplant powder) group or placebo (capsules containing dextrin powder) group, stratified by sex, age, and hospital SBP and DBP during the first visit. The characteristics of participants in each group are summarized in Table 1. Assignment of food was conducted through the use of block randomization method by using a computer at a third-party data center (Media Educational Center, Hokkaido Institute of Information Technology, Ebetsu, Hokkaido, Japan). Clinical research collaborators were blinded to the assignment information during the study period. The assignment information was opened after analysis data was finalized.

### 2.2. Power Analysis of Sample Size

We calculated that a sample size of 80 (40 in each group) was necessary using the effect size of 0.63 which was detected by the result of pilot study over an 8 week treatment period (registered at https://upload.umin.ac.jp/cgi-open-bin/ctr_e/ctr_view.cgi?recptno=R000032943; registration number, UMIN000028785; date of registration, August 13, 2017) with a statistical power of 80% and an α of 5%. Assuming a 20% loss in the follow-up rate, 100 participants (50 in each group) were enrolled.

### 2.3. Preparation of Eggplant Powder and Capsules

Eggplant powder was made from eggplant fruit juice containing excipient through dry powdering. The test food was defined as a daily dose of eggplant-derived choline ester (ACh). The amount of ACh was set as 2.3 mg/day.

Eggplants cultivated in Kochi prefecture (Japan) were used as raw materials. Fresh eggplant fruits were washed, cut into pieces, heated to prevent degeneration, and then squeezed to remove the pulp, generating eggplant juice. Dextrin was added to the juice, which was then lyophilized. This lot of lyophilized eggplant powder containing 1.92 mg/g of eggplant-derived choline ester (ACh) was obtained with a yield of 4.08% from the raw materials, and then encapsulated to afford 300 mg capsules. Placebo capsules contained only dextrin powder, without eggplant powder. The eggplant and placebo capsules were prepared under strict quality-controlled protocols and were identical in appearance. Analyses of the nutrient composition of eggplant and placebo capsules were conducted using the methods established by the Japan Food Research Laboratories (Tokyo, Japan), and the results are presented in Table 2. ACh was measured by using liquid chromatography-tandem mass spectrometry (LC-MS/MS) at Shinshu University, as described below. In the clinical trial, the participants received daily ingestion of 1200 mg eggplant powder (300 mg powder containing 25% dextrin × 4), which was produced from approximately 22 g of the fresh eggplant.

### 2.4. Treatment

This clinical trial was conducted at Hokkaido Information University, Health Information Science Research Center, Ebetsu, Hokkaido, Japan. The participants ingested four eggplant powder capsules containing 2.3 mg of ACh or four placebo capsules every day for 12 weeks. Instructions were to swallow two capsules in the morning (after breakfast) and two capsules in the evening (before sleep). Medical interviews, vital sign and body composition measurements, urinary assessments, hematological and biological assessments, and questionnaire completion were conducted during the baseline, week 4, week 8, week 12, and week 16 (four weeks after the end of ingestion). During the entire course of this study, participants were asked to maintain their daily activities, including food consumption and exercise habits, and to avoid consuming any supplements, eggplant, and bamboo shoot, including processed foods containing them, and they recorded their daily activities. Bamboo shoot is known to contain a similar level of ACh as eggplant, and the test food was the only ACh-rich food allowed to be consumed by the participants.

The primary outcome assessed was hospital BP comprising SBP and DBP. The secondary outcomes assessed were home BP (morning and evening), stress assessed by using the visual analogue scale (VAS) questionnaire, and PSs assessed by using the full-length version of the ‘Profile of Mood States 2nd Edition (POMS-2) for Adults’.

### 2.5. Quantitation of Hypotensive Ingredients in Eggplant Capsules

The quantitation of ACh was performed by the standard addition method according to the method described in our applied patent [21] with minor changes. A portion of the test food was added to (2-aminoethyl) trimethylammonium pivaloylamide (EN; internal standard, synthesized at Shinshu University) and then extracted with 50 mM hydrochloric acid (HCl) three times. The extract was mixed together and applied to a weak acidic cation exchange cartridge (Inertsep CBA 100 mg/1 mL; GL Science Inc., Tokyo, Japan). The fraction containing ACh was eluted with 1 M HCl and collected into a measuring flask. After being filled up to appropriate amount with the mobile phase, the solution was dispensed into three aliquots and spiked with different amount of standard ACh to make dilution series. ACh and EN in the samples were analyzed by LC-MS/MS with following condition. The separation was performed using isocratic mobile phase (33% (*v/v*) methanol containing 0.010% formic acid) on a YMC Triart-PFP column (4.6 mm × 250 mm, 5 µm; YMC. Co., Ltd., Kyoto, Japan). ACh and EN were detected by positive multiple reaction monitoring mode (MRM) at the following MRM transitions, 146.15 > 87.10 (ACh) and 187.30 > 128.15 (EN). The quantitative value of ACh was corrected by the recovery of EN. The quantitation of γ-aminobutyric acid (GABA) and chlorogenic acid (CA) was also performed in a similar manner at the following MRM transitions 104.15 > 87.20 (GABA) and 355.10 > 163.20 (CA).

### 2.6. Hospital BP and Home BP Assessments

Hospital BP was measured by a doctor or nurse using an Automatic Blood Pressure Monitor HEM-7080IC (Omron Healthcare Co., Ltd., Kyoto, Japan) using the upper arm of the nondominant arm after a >10-min rest. Three sequential measurements were performed, and the median of the measurements was taken at each evaluation point.

Home BP was measured by the participants using an Automatic Blood Pressure Monitor HEM-7080IC using the upper arm of the nondominant arm. Participants measured BP daily for one week prior to visits 2–6, within 1 h after waking up (morning BP), and before going to bed (night BP). Three sequential measurements were performed, and the median of the measurements was taken each day. The average BP during three days prior to each evaluation point was evaluated.

### 2.7. Physical, Hematological, Biochemical, and Urinary Tests

Blood was collected from the participants after a 12 h fasting and used for the following hematological examinations: white blood cell (WBC), red blood cell (RBC), hemoglobin (Hb), hematocrit (Hct), and blood platelet (Plt) count. Biological examinations included liver function (aspartate aminotransferase (AST), alanine aminotransferase (ALT), gamma-glutamyl transpeptidase (γ-GTP), alkaline phosphatase (ALP), and lactate dehydrogenase (LDH) levels); renal function (blood urea nitrogen (BUN), creatinine (CRE), and uric acid (UA) levels); lipid profiles (total cholesterol (TC), low-density lipoprotein cholesterol (LDL-C), high-density lipoprotein cholesterol (HDL-C), and triglyceride (TG) levels); and blood glucose profiles (fasting plasma glucose (FPG) and hemoglobin (Hb) A1c levels).

First void urine was collected, and the sample was used for qualitatively assessments of pH, sugar, protein, occult blood, urobilinogen, and ketones.

Blood and urine tests were analyzed at Sapporo Clinical Laboratory, Inc. (Hokkaido, Japan). Body composition (BW, body fat rate (BFR), and BMI) was assessed using a Body Composition Analyzer DC-320 (Tanita Corp, Tokyo, Japan).

### 2.8. VAS Questionnaire Assessing Stress and Profile of PSs by POMS-2

We used a VAS questionnaire to assess participants’ stress. Participants were instructed to place an “X” along a 100 mm line to provide a rating from the worst condition (the left end, 0 mm) to best condition (the right end, 100 mm) for each question based on their current health condition. The questionnaire results were assessed by evaluating the length from the beginning of the line on the left to the “X”, and the increase of score considered as improvement stress.

The POMS-2 questionnaire was used to evaluate the effects of eggplant on PSs. Participants were instructed to complete it prior to taking the capsules. Anger-hostility, confusion-bewilderment, depression-dejection, fatigue-inertia, and tension-anxiety were defined as negative PSs, whereas vigor-activity and friendliness were defined as positive PSs. All PSs were assessed using the POMS-2 full version for adults, which comprised 65 questions (Success Bell, Tokyo, Japan). Participants selected one of five answers ranging from “not at all” to “quite a lot” based on their PS over the previous week. Total mood disturbance scores, which were comprehensively expressed as negative PSs, were calculated by adding anger-hostility, confusion-bewilderment, depression-dejection, fatigue-inertia, and tension-anxiety scores, and then subtracting the vigor-activity and friendliness scores.

For assessing the antistress effects of eggplant, participants first ingested two capsules and then completed the Uchida–Kraepelin Psychodiagnostic Test (UKT) for 15 min to induce mental stress [22]. The VAS questionnaire was completed two times: before intake of test food and after completion of the UKT.

### 2.9. FFQg

In addition, all participants completed a Food Frequency Questionnaire Based on Food Groups (FFQg) (Kenpakusha, Tokyo, Japan) during the second to sixth visits. The FFQg was used to estimate nutrient (calories, protein, fat, carbohydrates, dietary fiber, and salt) intake based on the participants’ regular diet. Participants reported their weekly amount and frequency of food intake about 29 food groups and 10 types of cooking methods.

### 2.10. Ethics

The current clinical study was conducted in accordance with the Declaration of Helsinki and the ethical guidelines on medical research with humans (Ministry of Education, Culture, Sports, Science and Technology, and Ministry of Health, Labor and Welfare). Written informed consent was obtained from participants after reading a study consent form of clinical trial prior to being enrolled. The study protocol was approved by the ethics committee of Hokkaido Information University (Ebetsu, Hokkaido, Japan; approved on 25 May 2018; approval number: 2018–06). This study is registered at https://upload.umin.ac.jp/cgi-open-bin/ctr_e/ctr_view.cgi?recptno=R000037995 (registered on 10 July 2018; registration number: UMIN000033330).

### 2.11. Statistical Analysis

Student’s *t*-test was used to analyze the primary and secondary outcomes, safety outcomes, and food frequency questionnaire values by comparing the changes in participant values between the two groups. Changes in participant values were analyzed using repeated measures of analysis of variance between groups. For participant characteristics, Fisher’s exact probability test was used for sex and the Mann–Whitney U test was used for intake rate; Student’s *t*-test was used for other participant characteristics. In a subgroup analysis, we analyzed hospital BP in participants with SBP of 130–139 mmHg or DBP of 85–89 mmHg (normal-high BP participants) and those with SBP of 140–159 mmHg or DBP of 90–99 mmHg (grade1 hypertension participants). All statistical analyses were performed using SPSS v25 (IBM Japan, Ltd., Tokyo, Japan). Exploratory data analysis using measured values were also performed in the same manner. All results are expressed as the mean ± standard error. It was considered a statistically significant difference when *p-*value was less than 0.05.

## 3. Results

### 3.1. Participant Dropouts and Characteristics

**Figure 1:**
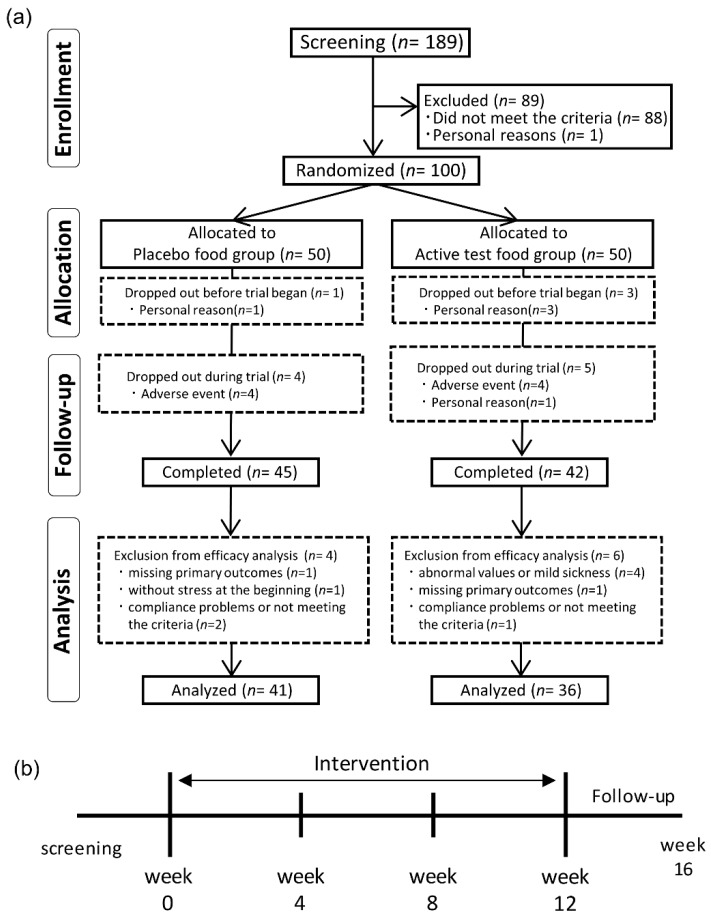
Flowcharts of (**a**) participant election and (**b**) study schedule.

Participant involvement throughout the study period and study schedule are shown in Figure 1. Volunteers who provided informed consent (*n* = 189) were assessed for eligibility, and after screening, 100 participants were enrolled in this study. All enrolled participants were randomized into one of two intervention groups (placebo group, *n* = 50; eggplant group, *n* = 50). Four participants (personal reason, *n* = 4) dropped out prior to study initiation, and nine participants dropped out during the study for the adverse events or personal reason (placebo group, adverse event such as pneumonia (*n* = 1), colonic diverticulitis (*n* = 1), dyslipidemia (*n* = 1), and hives (*n* = 1); eggplant group, adverse event such as injury (*n* = 2), mild liver dysfunction (*n* = 1) and nephropathy (*n* = 1), personal reason (*n* = 1)). Finally, 87 participants completed this study: 42 in the eggplant group and 45 in the placebo group. Ninety-six participants, excluding the four participants who dropped out because of personal reasons before the start of the study, were included in the safety analysis. We excluded 10 participants in the efficacy analysis because of abnormal variation (outside of the reference range (*n* = 1), and >120% changes compared to the baseline among safety outcomes (*n* = 1)) in values or mild sickness (allergies unrelated to the test food (*n* = 2)), compliance problems, or participant not meeting the criteria (*n* = 3), without stress at the beginning of treatment (*n* = 1), missing primary outcomes owing to the absence of BP measurement (*n* = 2). The efficacy analysis comprised of 36 participants in the eggplant group and 41 in the placebo group. Sex ratio, mean age, height, BMI, hospital SBP, hospital DBP, and intake rate for each group are shown in Table 1. There were no differences between the two groups regarding these characteristics.

### 3.2. Efficacy of Eggplant on Hospital BP

**Figure 2:**
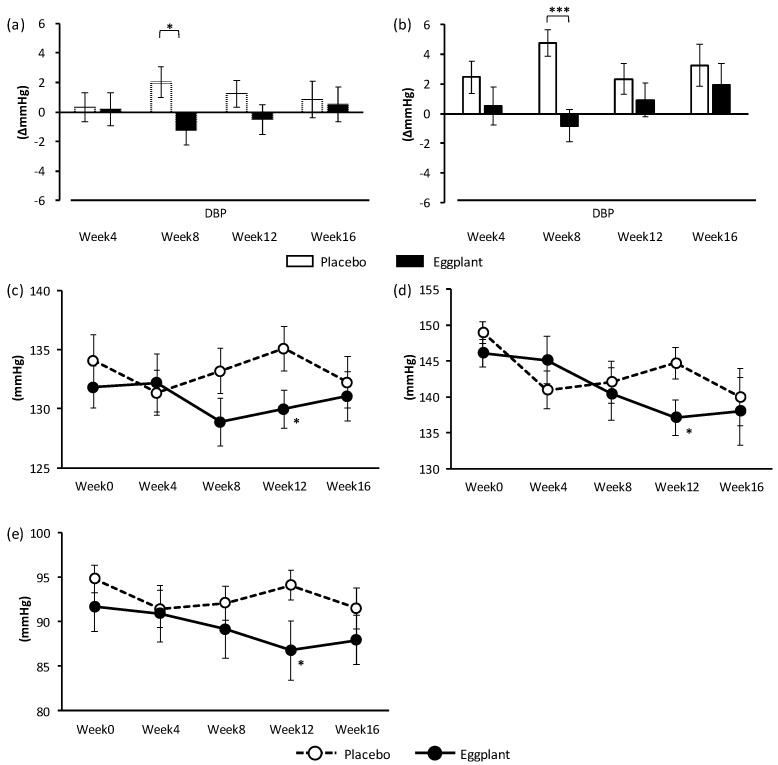
Changes in hospital BP (different values from week 0: (**a**) and (**b**); measured values: (**c**), (**d**), and (**e**)). (**a**) DBP of all participants. (**b**) DBP of participants with normal-high BP. (**c**) SBP of all participants, (**d**) SBP of participants with grade 1 hypertension, (**e**) DBP of participants with grade 1 hypertension. Student’s *t*-test was conducted for data analysis. Statistical significance: * *p* < 0.05 and *** *p* < 0.001 vs. placebo group. Values are presented as mean ± standard error. BP: blood pressure; DBP: diastolic blood pressure; SBP: systolic blood pressure.

Eggplant powder intake significantly improved DBP at week 8, compared to that in the placebo group (*p* = 0.024) (Figure 2a, Table S1). In a subgroup analysis, the DBP increase was significantly suppressed at week 8 (*p* < 0.001) following ingestion of eggplant in participants with normal-high BP (placebo: *n* = 26; eggplant: *n* = 27) (Figure 2b, Table S1). Exploratory data analysis showed significantly lower SBP at week 12 in the eggplant group than in the placebo group (*p* = 0.046) (Figure 2c, Table S2). The SBP and DBP of participants with grade 1 hypertension (placebo: *n* = 15; eggplant: *n* = 9) were significantly lower at week 12 in the eggplant group than in the placebo group (SBP: *p* = 0.037, DBP: *p* = 0.041) (Figure 2d, e, Table S2). Moreover, the significance of the time–food interaction of hospital SBP overall (change and measured value, *p* = 0.018) and the grade 1 hypertension group (change and measured value, *p* = 0.043) and hospital DBP in the normal-high BP group (change and measured value, *p* = 0.028) also supported the BP lowering effect of the eggplant powder ingestion (Tables S1 and S2).

### 3.3. Efficacy of Eggplant on Home BP

**Figure 3:**
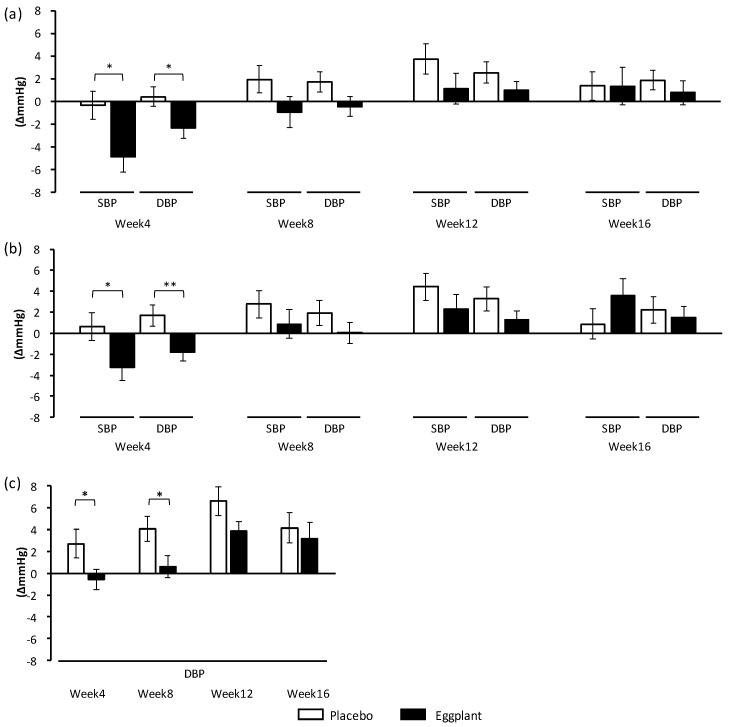
Changes in home BP (different values from week 0). (**a**) Morning SBP and DBP of all participants, (**b**) Morning SBP and DBP of participants with normal-high BP, (**c**) evening DBP of participants with normal-high BP. Student’s *t*-test was conducted for data analysis. Statistical significance: * *p* < 0.05 and ** *p* < 0.01 vs. placebo group. Values are presented as mean ± standard error. BP: blood pressure; DBP: diastolic blood pressure; SBP: systolic blood pressure.

Significant effects of eggplant powder on home BP (different values from week 0) are shown in Figure 3. Intake of eggplant powder significantly decreased morning SBP (Figure 3a, *p* = 0.017) and DBP (Figure 3a, *p* = 0.032) at week 4, compared to those in the placebo group. In addition, subgroup analyses showed a significant decrease in morning SBP (Figure 3b, *p* = 0.041) and DBP (Figure 3b, *p* = 0.008) at week 4 and significant suppression in the evening DBP increase at week 4 (Figure 3c, *p* = 0.044) and week 8 (Figure 3c, *p* = 0.029) in normal-high BP participants in the eggplant group compared to those in the placebo group. There was no significant difference in the measured values between the placebo and eggplant groups.

### 3.4. Efficacy of Eggplant on Stress and PSs

**Figure 4:**
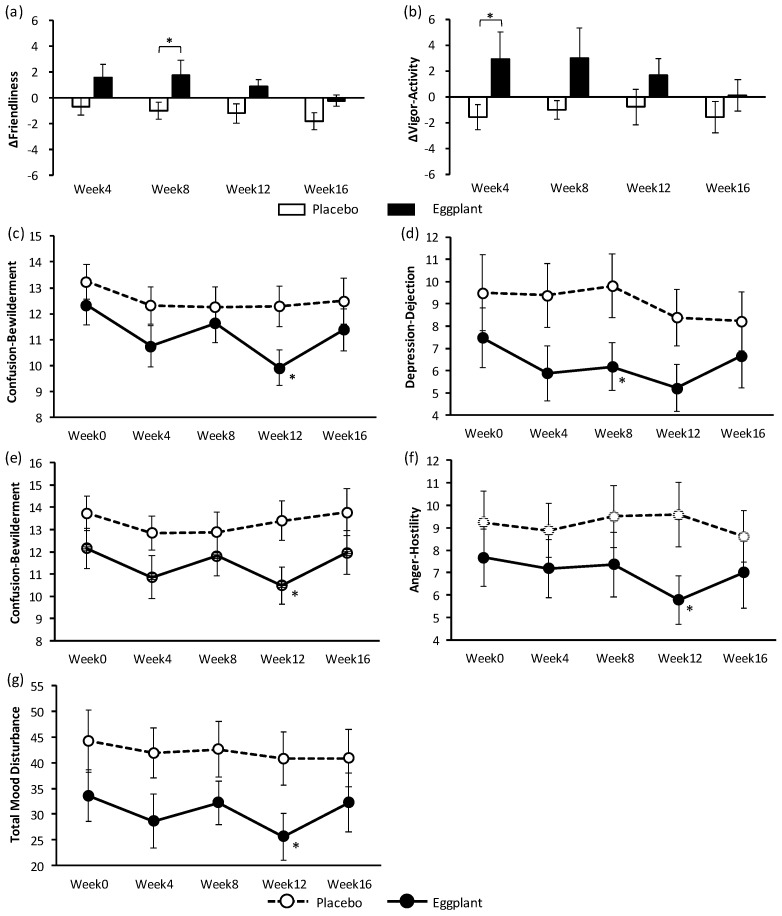
Changes in POMS-2 scores (different values from week 0: (**a**) and (**b**); measured values: (**c**), (**d**), (**e**), (**f**), and (**g**)). (**a**) Friendliness and (**b**) vigor-activity of participants with grade 1 hypertension. (**c**) Confusion-Bewilderment scores of all participants. (**d**) Depression-dejection, (**e**) confusion-bewilderment, (**f**) anger-hostility, and (**g**) total mood disturbance in participants with normal-high BP. * *p* < 0.05 vs. placebo group. Values are presented as mean ± standard error. BP: blood pressure; POMS-2: Profile of Mood States 2nd Edition.

To assess the effects of eggplant on stress and PSs, changes in VAS scores, which indicated stress, and POMS-2 scores, which indicated PSs, were evaluated (Tables S3 and S4). There were no differences in VAS scores between the eggplant and placebo groups, both before and after UKT. Subgroup analyses showed that the difference in POMS-2 scores from week 0, ‘vigor-activity’ at week 4, and ‘friendliness’ at week 8 of participants with grade 1 hypertension in the eggplant group were significantly superior to those in the placebo group (Figure 4). Exploratory data analysis showed significant improvement in ‘confusion-bewilderment’ in all participants at week 12 and negative PSs (‘depression-dejection’ at week 8; ‘confusion-bewilderment’, ‘anger-hostility’, and ‘total mood disturbance’ at week 12) in participants with normal-high BP in the eggplant group (Figure 4).

### 3.5. Assessment of Dietary Nutrients of Participants During the Study

For the assessment of dietary nutrients, the participants completed FFQg (Table S5). Lipid and dietary fibers at week 12 in the eggplant group were significantly lower than those in the placebo group (*p* = 0.009 and 0.035, respectively). However, there were no statistically significant differences in the intake of calories, proteins, carbohydrates, and salt between the eggplant and placebo groups.

### 3.6. Assessment of Safety of Eggplant

To analyze the safety of eggplant, we evaluated the body composition, complete blood count, liver function, renal function, lipid profiles, and blood glucose profiles of participants (Table S6). Additionally, we qualitatively assessed urinary parameters (Table S6). All changes in the values were within the reference value ranges. Adverse effects were observed in each group. However, the principal investigator inferred that all adverse effects were not related to the test food according to the protocol criteria. Thus, the eggplant powder intake had no or minimal unfavorable effects, at the raw eggplant dose of 22 g/day, which contained 2.3 mg of eggplant-derived choline ester (ACh).

## 4. Discussion

The randomized, double-blind, placebo-controlled, parallel group comparison study showed that continuous intake of eggplant powder containing 2.3 mg/day of eggplant-derived choline ester (ACh) improved BP in participants with normal-high BP and grade 1 hypertension. Subgroup analysis confirmed the effect of the eggplant powder on normal-high BP participants. A thorough search of the literature confirmed that this is the first clinical trial providing evidence of the beneficial effects of eggplant on BP. In addition, this study showed that eggplant intake improved positive and negative PSs.

Hospital DBP, which is the primary outcome in this study, was improved overall and specifically in the normal-high BP group. Interestingly, the improvement in DBP upon eggplant intake was more significant in the normal-high BP group (*p* < 0.001) than overall (*p* = 0.024). Improvements in hospital SBP overall and in the grade 1 hypertension group, as well as hospital DBP in the grade 1 hypertension group, were also shown through exploratory data analysis. Eggplant intake improved DBP in the normal-high BP group, and SBP and DBP in the grade 1 hypertension group, indicating that eggplant exerted different BP-improving effects for different BP categories.

The secondary outcomes, home SBP and DBP, were also improved by eggplant intake. The morning SBP and DBP overall and in the normal-high BP group, as well as the evening DBP of the normal-high BP group were also improved, supporting the hospital BP-improving effects of the eggplant intake. Management of morning BP is important in heart failure prevention [23,24]. It has also been reported that morning and night BPs of patients with anxiety disorder are significantly higher than those of healthy participants [25,26], and one of the causes was estimated to be sleeping disorder due to sympathetic overactivity [27]. Thus, we speculated that eggplant intake suppressed sympathetic nervous activity in the participants during sleep, thereby improving the morning BPs of participants with stress.

Previously, we reported the BP-lowing effect of eggplant in SHRs [16]. Daily oral intake of low-dose eggplant powder (0.821 mg/kg BW) significantly prevented increases in both the SBP and DBP of SHRs, and the effective dose of the eggplant powder for an adult person (60 kg) was calculated to be 13.1 mg/day by Kleiber’s law [28]. The efficacy of ACh in eggplant may be lower in human participants with normal-high BP and grade 1 hypertension than in SHRs that developed high-grade hypertension. In the SHR study, urinary catecholamine level was decreased by ACh in eggplant by acting on the M3 muscarinic ACh receptor (M3 mAChR), suggesting that ACh in eggplant lowers BP by suppressing sympathetic nervous activity through M3 mAChR in the digestive system [16]. M3 mAChR is present on epithelial cells of the stomach and intestine [29], and orally administrated eggplant-derived ACh may act on it to stimulate the parasympathetic nervous system. This stimulation travels to the medulla oblongata, where sympathetic nervous activity is automatically reduced due to reciprocal innervation. Abnormal neuromodulation is considered a cause of BP elevation in SHRs. Blood catechol amine level in SHRs was reported to be higher than that in normotensive Wister Kyoto (WKY) rats, which was attributed to abnormal acceleration of sympathetic nervous activity in SHRs [30,31]. The sensitivity of the baroreceptor reflex function in SHRs was also reported to be lower than that in WKY rats [32]. It was suggested that neuromodulation to control BP varied among BP categories, and ACh in eggplant reduced BP more efficiently in participants with grade 1 hypertension versus those with normal high BP. Regarding the primary outcome, hospital BP, eggplant intake improved only DBP in normal-high BP participants, but it improved both SBP and DBP in grade 1 hypertension participants. The difference in BP between the eggplant and placebo groups was −5.6 mmHg for DBP in the normal-high BP participants, and −7.6 mmHg for SBP and −7.3 mmHg for DBP in the grade 1 hypertension participants.

Stress and negative PSs increase sympathetic nervous activity; thus, suppression of sympathetic nervous activity could reduce stress and improve PSs. Therefore, participants with stress were employed in the study to evaluate the effect of eggplant on stress and PSs using VAS and POMS-2 questionnaires, respectively. A very simple VAS questionnaire was used to evaluate stress after oral intake tests in another work [33]. PSs are reported to be involved in the development and state of hypertension [34,35], and the POMS-2 test was therefore used to assess PSs [36]. Results of the VAS and POMS-2 tests indicated that long-term intake of eggplant powder had no effect on stress but improved PSs. The antistress effect of eggplant should be further examined with an objective method instead of the subjective assessment with the VAS questionnaire. In the POMS-2 test, eggplant intake improved negative PSs (‘confusion-bewilderment’ of all participants and ‘depression-dejection’; ‘confusion-bewilderment’, ‘anger-hostility’, and ‘total mood disturbance’) in the normal-high BP group and positive PSs (‘friendliness’ and ‘vigor-activity’) in the grade 1 hypertension group. The results of the POMS-2 test also indicated different efficiency of eggplant for participants in different BP categories. The trend of improvement in the PS from week 0 to week 4 was suggested to be an effect of acclimatization. At week 8, PSs seemed to deteriorate; this may be because of an earthquake that occurred on September 6, 15 days prior to the week 8 examination, although there was no impact on the test overall.

Dietary intervention in this study included the intake of eggplant powder capsules and avoidance of daily ACh-rich foods, including eggplant and bamboo shoot. The daily dose of eggplant powder in the treatment group capsules was 1.2 g, which contained 4.39 kcal. The placebo capsules also contained 4.35 kcal, without the eggplant powder. Therefore, we concluded that the observed BP- and PS-improving effects were due to the test food. In addition to ACh, eggplant contains GABA [37] and CA [38], which are compounds with hypotensive effect. The effective doses of GABA and CA (derived from coffee) for antihypertensive effect in adult individuals are 12.3 mg/day and 271 mg/day, respectively, according to the “Foods with Function Claims” in Japan. The test food in this study contained 7.65 mg/day of GABA (62.2% of the effective dose) and 12.3 mg/day of CA (4.54% of the effective dose), which were both lower than the respective effective doses. We have reported that eggplant-derived ACh, not GABA or CA, is the main compound responsible for the hypotensive effects of eggplant in SHRs [16]. This result should be applied when considering the active ingredient in eggplant to reduce BP in a clinical trial of participants with relatively higher blood pressure. Both GABA and CA in the test food were estimated to be ineffective alone because of the low dose. No data indicating a synergistic effect enhancing the individual hypotensive effects have been reported. It is reasonable to assume that the antihypertensive effect was from the ACh. Therefore, the BP-improving effects of GABA and CA in the test food should be limited in this study, and the main antihypertensive component in the eggplant powder was concluded to be ACh. A study on the antihypertensive effect of orally administered ACh is currently being conducted, and we expect positive results. This randomized controlled trial confirmed the safety of eggplant powder at a dose equivalent to 22 g/day of raw eggplant.

In conclusion, eggplant powder intake significantly improved BP and PSs, compared with those in the control group. Changes in hospital and home BPs overall and in the normal-high BP group were transiently improved at week 4 and 8. The reasons were estimated to be environmental factors. It is well-known that BP, including hospital and home BP, varies by the season [39]. From summer to winter, BP increases with decreasing temperature. The clinical trial was conducted from the end of summer to early winter, and the average temperature dropped from approximately 27.9 °C to 9.6 °C, corresponding with an estimated 3.5 mmHg increase in BP [39]. The increase in BP due to temperature decrease and the BP-lowering effect of eggplant powder intake could be mediated through neuromodulation, and both effects may have competed against each other, thus attenuating the effect of eggplant after 4 or 8 weeks from beginning of the test. The measured hospital BPs overall and in the grade 1 hypertension group were also transiently improved at week 12, which should be caused by prolonged intake of eggplant powder. These characteristic effects of eggplant powder seemed to be mild and gradual, which was different from the effects of current antihypertensives. Therefore, continuous intake of eggplant is expected to improve BP in a person with normal-high BP or grade 1 hypertension. Eggplant is recognized as safe. This clinical trial confirmed the safety as well as the BP and PSs improving effects of eggplant powder. These novel effects were expected to support the use of the ACh-containing eggplant powder as a safe treatment for BP and mental health.

## 5. Conclusions

A randomized, double-blind, placebo-controlled, parallel-group comparative study showed that ingestion of eggplant powder for 12 weeks improved BP and PSs without side effect. The eggplant powder dose was 1.2 g/day contained 2.3 mg of eggplant-derived choline ester (ACh), which was a novel functional food factor. This was the first evidence of the antihypertensive and PS-improving effects of eggplant. Eggplant is expected to be utilized as a safe treatment for BP and mental health.

## Figures and Tables

**Table 1:** Characteristics of participants and intake rates of test foods in the eggplant and placebo groups.

Characteristic	Unit	Placebo	Eggplant	*p*
Subjects	n	41	36	–
Male	n	16	9	0.228
Age	years	54.4 ± 6.2	54.2 ± 7.2	0.886
Height	cm	163.1 ± 8.4	159.6 ± 7.5	0.065
Body mass index	kg/m^2^	22.9 ± 2.7	22.5 ± 3.2	0.596
Visual analogue scale	mm	42.4 ± 18.5	41.9 ± 16.3	0.901
Hospital systolic blood pressure	mmHg	138.3 ± 8.8	137.1 ± 7.8	0.518
Hospital diastolic blood pressure	mmHg	86.1 ± 8.5	85.0 ± 7.9	0.578
Intake rate	%	99.0 ± 1.8	98.9 ± 1.8	0.947

**Table 2:** Nutrient composition of the eggplant and placebo capsules administered per day.

Composition	Placebo	Eggplant
Calories (kcal)	4.35	4.39
Water (g)	0.03	0.03
Proteins (g)	0.00	0.11
Lipids (g)	0.00	0.02
Carbohydrates (g)	1.09	0.95
Ash (g)	0.00	0.09
Sodium (mg)	0.00	0.30
Choline ester (Acetylcholine, mg)	0.00	2.30
γ-Aminobutyric acid (GABA, mg)	0.00	7.65
Chlorogenic acid (CA, mg)	0.00	12.3

## Supplementary Materials

The following are available online at https://www.mdpi.com/2072-6643/11/11/2797/s1, Table S1: Changes in blood pressure from week 0; Table S2: Blood pressure (measured value); Table S3: VAS scores indicating stress and POMS-2 scores indicating psychological state (measured value); Table S4: Changes in VAS and POMS-2 scores from week 0; Table S5: Dietary nutrients consumed by participants during the study; Table S6: Body composition, complete blood count, liver function, renal function, lipid profiles, blood glucose profiles, and urinary tests.

## References

[1] World Health Organization (2013). A Global Brief on Hypertension: Silent killer, Global Public Health Crisis. https://www.who.int/cardiovascular_diseases/publications/global_brief_hypertension/en/.

[2] Chobanian A.V., Bakris G.L., Black H.R., Cushman W.C., Green L.A., Izzo J.L., Jones D.W., Materson B.J., Oparil S., Wright J.T. (2003). The National High Blood Pressure Education Program Coordinating Committee. The seventh report of the joint national committee on prevention, detection, evaluation, and treatment of high blood pressure. JAMA.

[3] Go A.S., Bauman M.A., King S.M.C., Fonarow G.C., Lawrence W., Williams K.A., Sanchez E. (2014). An effective approach to high blood pressure control: A science advisory from the American Heart Association, the American College of Cardiology, and the Centers for Disease Control and Prevention. J. Am. Coll. Cardiol..

[4] Williams B., Poulter N.R., Brown M.J., Davis M., McInnes G.T., Potter J.F., Sever P.S., Thom S.M. (2004). British Hypertension Society. Guidelines for management of hypertension: Report of the fourth working party of the British Hypertension Society, 2004–BHS IV. J. Hum. Hypertens..

[5] Mohamed S. (2014). Functional foods against metabolic syndrome (obesity, diabetes, hypertension and dyslipidemia) and cardiovascular disease. Trends Food Sci. Tech..

[6] United States Department of Agriculture Agricultural Research Service (USDA ARS) National Nutrient Database for Standard Reference Release 28 (https://ndb.nal.usda.gov/ndb/). https://ndb.nal.usda.gov/ndb/foods/show/2962?fgcd=&manu=&lfacet=&format=&count=&max=50&offset=&sort=default&order=asc&qlookup=eggplant&ds=&qt=&qp=&qa=&qn=&q=&ing=/.

[7] National Institute of Diabetes and Digestive and Kidney Diseases (NIDDK), National Institutes of Health (NIH) Nutrition Research Report 2015 & 2016. Preventing type 2 diabetes. https://www.niddk.nih.gov/health-information/diabetes/overview/preventing-type-2-diabetes/.

[8] American Diabetes Association (ADA) Eating colorful food has health benefits. http://www.diabetesforecast.org/2011/aug/eating-colorful-food-has-health-benefits.html.

[9] American Diabetes Association (ADA) Food for your plate. http://www.diabetesforecast.org/2015/adm/diabetes-plate-method/foods-for-your-plate.html.

[10] Guimarães P.R., Galvão A.M.P., Batista C.M., Azevedo G.S., Oliveira R.D., Lamounier R.P., Freire N., Barros A.M.D., Sakurai E., Oliveira J.P. (2000). Eggplant (*Solanum melongena*) infusion has a modest and transitory effect on hypercholesterolemic subjects. Braz. J. Med. Biol. Res..

[11] Scorsatto M., Rosa G., Luiz R.R., Mulder A.R.P., Teodoro A.J., Oliveiram G.M.M. (2019). Effect of eggplant flour (*Solanum melongena* L.) associated with hypoenergetic diet on antioxidant status in overweight women—A randomised clinical trial. Int. J. Food Sci. Techol..

[12] Horiuchi Y., Kimura R., Kato N., Fujii T., Seki M., Endo T., Kato T., Kawashima K. (2003). Evolutional study on acetylcholine expression. Life Sci..

[13] Kawashima K. (2010). Origin of acetylcholine and expression of non-neuronal acetylcholine. Biomed. Gerontol..

[14] Nakamura K., Naramoto K., Koyama M. (2013). Blood-pressure-lowering effect of fermented buckwheat sprouts in spontaneously hypertensive rats. J. Funct. Foods.

[15] Nakamura K., Okitsu S., Ishida R., Tian S., Igari N., Amano Y. (2016). Identification of natural lactoylcholine in lactic acid bacteria-fermented food. Food Chem..

[16] Yamaguchi S., Matsumoto K., Koyama M., Tian S., Watanabe M., Takahashi A., Miyatake K., Nakamura K. (2019). Antihypertensive effects of orally administered eggplant (*Solanum melongena*) rich in acetylcholine on spontaneously hypertensive rats. Food Chem..

[17] Molitor H. (1936). A comparative study of the effects of five choline compounds used in therapeutics: Acetylcholine chloride, acetyl beta-methylcholine chloride, carbaminoyl choline, ethyl ether beta-methylcholine chloride, carbaminoyl beta-methylcholine chloride. J. Pharmacol. Exp. Ther..

[18] Wilson M.A., Fadel J.R. (2017). Cholinergic regulation of fear learning and extinction. J. Neurosci. Res..

[19] Balkan B., Pogun S. (2018). Nicotinic cholinergic system in the hypothalamus modulates the activity of the hypothalamic neuropeptides during the stress response. Curr. Neuropharmacol..

[20] Steptoe A. (2000). Psychosocial factors in the development of hypertension. Ann. Med..

[21] Nakamura K. (2018). Choline Ester-Containing Composition for Oral Ingestion. Patent.

[22] Sugimoto K., Kanai A., Shoji N. (2009). The effectiveness of the Uchida-Kraepelin test for psychological stress: An analysis of plasma and salivary stress substances. Biopsychosoc. Med..

[23] White W.B. (2007). Clinical assessment of early morning blood pressure in patients with hypertension. Prev. Cardiol..

[24] Wang J.G., Kario K., Chen C.H., Park J.B., Hoshide S., Huo Y., Lee H.Y., Li Y., Mogi M., Munakata M. (2018). Management of morning hypertension: A consensus statement of an Asian expert panel. J. Clin. Hypertens..

[25] Kayano H., Koba S., Matsui T., Fukuoka H., Toshida T., Sakai T., Akutsu Y., Tanno K., Geshi E., Kobayashi Y. (2012). Anxiety disorder is associated with nocturnal and early morning hypertension with or without morning surge: Ambulatory blood pressure monitoring. Circ. J..

[26] Mellman T.A., Brown D.D., Jenifer E.S., Hipolito M.M.S., Randall O.S. (2019). Posttraumatic stress disorder and nocturnal blood pressure dipping in young adult African-Americans. Psychosom. Med..

[27] Veith R.C., Lewis N., Linares O.A., Barnes R.F., Raskind M.A., Villacres E.C., Murburg M.M., Ashleigh E.A., Castillo S., Peskind E.R. (1994). Sympathetic nervous system activity in major depression: Basal and desipramine-induced alterations in plasma norepinephrine kinetics. Arch. Gen. Psychiatry..

[28] Kleiber M. (1961). The Fire of Life: An Introduction to Animal Energetics.

[29] Wess J. (2004). Muscarinic acetylcholine receptor knockout mice: Novel phenotypes and clinical implications. Ann. Rev. Pharmacol. Toxicol..

[30] Tsunoda M., Takezawa K., Ina Y., Nagashima K., Ohomori K., Kobayashi S., Imai K. (2000). New approach for measurement of sympathetic nervous abnormality in conscious, spontaneously hypertensive rats. Jpn. J. Pharmacol..

[31] Donohue S.J., Stitzel R.E., Head R.J. (1988). Time course of changes in the norepinephrine content of tissues from spontaneously hypertensive and Wistar Kyoto rats. J. Pharmacol. Exp. Ther..

[32] Kumagai H., Averill D.B., Ferrario C.M. (1992). Renal nerve activity in rats with spontaneous hypertension: Effect of converting enzyme inhibitor. Am. J. Physiol..

[33] Diaper A., Papadopoulos A., Rich A.S., Dawson G.R., Dourish C.T., Nutt D.J., Bailey J.E. (2012). The effect of a clinically effective and non-effective dose of lorazepam on 7.5% CO_2_-induced anxiety. Hum. Psychopharmacol..

[34] Andrew M.J., Baker R.A., Kneebone A.C., Knight J.L. (2000). Mood state as a predictor of neuropsychological deficits following cardiac surgery. J. Psychosom. Res..

[35] Hassan K., Elimeleh Y., Shehadeh M., Fadi H., Rubinchik I. (2018). The relationship between hydration status, male sexual dysfunction and depression in hemodialysis patients. Ther. Clin. Risk Manag..

[36] Yu B.H., Dimsdale J.E., Mills P.J. (1999). Psychological states and lymphocyte beta-adrenergic receptor responsiveness. Neuropsychopharmacology.

[37] Horie H., Ando S., Saito T. (2013). The contents of γ-amino butyric acid in eggplant and its accumulation with heat treatment. J. Jpn. Soc. Food Sci..

[38] Singh A.P., Luthria D., Wilson T., Vorsa N., Singh V., Banuelos G.S., Pasakdee S. (2009). Polyphenols content and antioxidant capacity of eggplant pulp. Food Chem..

[39] Lewington S., Li L., Sherliker P., Guo Y., Millwood L., Bian Z., Whitlock G., Yang L., Collins R., Chen J. (2012). Seasonal variation in blood pressure and its relationship with outdoor temperature in 10 diverse regions of China: The China Kadoorie Biobank. J. Hypertens..

