# Supplementary material for: Daily Ingestion of Eggplant Powder Improves Blood Pressure and Psychological State in Stressed Individuals: A Randomized Placebo-Controlled Study
Source: Nutrients. 2019 Nov 16;11(11):2797. doi: 10.3390/nu11112797 (PMC6893753; doi:10.3390/nu11112797)
Supplement: Supplementary file 1 [file nutrients-11-02797-s001.pdf]

Table S1. Changes in blood pressure from week 0

		Week 0	ΔWeek 4	ΔWeek 8	ΔWeek 12	ΔWeek 16	*time x food interaction, p*[b]
Hospital SBP (mmHg)	Placebo	134.1±14.0	-2.7±11.0	-0.9±11.8	1.0±11.5	-1.9±12.9	0.018*
	Eggplant	131.8±10.6	0.4±13.0	-2.9±10.1	-1.9±9.3	-0.8±12.4	
	p[a]	0.43	0.26	0.42	0.24	0.70	
Hospital DBP (mmHg)	Placebo	83.5±10.4	0.3±6.3	2.0±6.5	1.2±5.8	0.9±8.0	0.13
	Eggplant	83.0±8.5	0.2±6.6	-1.3±5.9	-0.5±6.0	0.5±7.0	
	p[a]	0.80	0.93	0.024*	0.20	0.85	
Hospital SBP in participants with normal-high BP (mmHg)	Placebo	125.5±9.1	0.3±12.1	2.6±12.2	4.0±12.4	2.3±11.8	0.14
	Eggplant	127.1±6.8	0.8±14.2	-2.0±10.0	0.5±8.8	1.7±11.5	
	p[a]	0.48	0.90	0.14	0.24	0.86	
Hospital DBP in participants with normal-high BP (mmHg)	Placebo	77.0±5.6	2.5±5.6	4.8±4.7	2.3±5.3	3.3±7.3	0.043*
	Eggplant	80.1±6.4	0.5±6.7	-0.8±5.7	0.9±5.9	2.0±7.3	
	p[a]	0.069	0.26	p<0.001**	0.36	0.52	
Hospital SBP in participants with grade 1 hypertension (mmHg)	Placebo	149.0±5.9	-8.1±6.0	-6.9±8.2	-4.3±7.6	-9.0±12.0	0.028*
	Eggplant	146.1±5.8	-1.0±8.8	-5.7±10.6	-9.0±6.9	-8.1±12.9	
	p[a]	0.26	0.055	0.75	0.14	0.87	
Hospital DBP in participants with grade 1 hypertension (mmHg)	Placebo	94.8±5.9	-3.4±6.0	-2.7±6.7	-0.7±6.2	-3.3±7.5	0.079
	Eggplant	91.7±8.5	-0.8±6.6	-2.6±6.5	-4.9±4.2	-3.8±3.3	
	p[a]	0.30	0.33	0.95	0.089	0.84	
Home SBP in the morning (mmHg)	Placebo	131.5±12.7	-0.3±7.7	2.0±7.7	3.7±8.7	1.4±8.0	0.54
	Eggplant	133.6±14.1	-4.8±8.4	-0.9±8.2	1.1±8.3	1.3±9.9	
	p[a]	0.49	0.017*	0.12	0.18	0.98	
Home DBP in the morning (mmHg)	Placebo	81.2±10.6	0.4±5.6	1.7±5.7	2.6±6.0	1.9±5.5	0.54
	Eggplant	83.1±9.8	-2.3±5.4	-0.4±5.2	1.0±4.7	0.8±6.4	
	p[a]	0.44	0.032*	0.087	0.22	0.41	
Home SBP in the morning in participants with normal-high BP (mmHg)	Placebo	127.1±11.0	0.6±6.8	2.8±6.5	4.4±2.3	0.9±7.3	0.54
	Eggplant	129.6±12.1	-3.2±6.6	0.9±7.1	2.3±7.4	3.6±8.5	
	p[a]	0.44	0.041*	0.32	0.27	0.22	
Home DBP in the morning in participants with normal-high BP (mmHg)	Placebo	77.3±9.4	1.7±5.1	1.9±6.0	3.3±5.8	2.2±6.4	0.31
	Eggplant	81.3±8.4	-1.8±4.2	0.0±5.2	1.3±4.1	1.5±5.5	
	p[a]	0.12	0.008**	0.22	0.16	0.64	
Home SBP in the morning in participants with grade 1 hypertension (mmHg)	Placebo	139.0±12.2	-2.0±9.2	0.5±9.5	2.6±11.6	2.3±9.3	0.81
	Eggplant	145.4±13.7	-9.6±11.6	-6.4±9.0	-2.4±10.3	-5.4±11.3	
	p[a]	0.24	0.09	0.09	0.31	0.09	
Home DBP in the morning in participants with grade 1 hypertension (mmHg)	Placebo	88.0±9.2	-1.8±5.9	1.4±5.2	1.3±6.3	1.3±3.4	0.70
	Eggplant	88.4±12.2	-3.9±8.2	-1.8±5.2	0.1±6.5	-1.3±8.6	
	p[a]	0.91	0.48	0.16	0.68	0.41	
Home SBP in the evening (mmHg)	Placebo	126.7±11.9	0.0±8.4	4.0±8.5	6.3±11.1	5.7±11.1	0.88
	Eggplant	127.8±12.1	-0.9±7.1	2.1±7.3	4.7±9.0	3.7±9.9	
	p[a]	0.69	0.64	0.31	0.51	0.41	
	Placebo	75.8±9.7	0.4±6.8	2.8±6.0	5.2±6.7	3.3±6.9	0.76
	Eggplant	77.1±8.9	-0.5±4.9	1.0±5.0	3.5±5.6	3.0±7.2	

Home DBP in the evening (mmHg)	p^a	0.54	0.51	0.15	0.24	0.85	
Home SBP in the evening in participants with normal-high BP (mmHg)	Placebo	122.7±10.8	2.5±8.4	3.8±8.1	8.1±10.8	6.9±11.8	0.86
	Eggplant	124.2±10.6	-0.8±6.6	1.7±7.3	5.2±7.2	4.4±10.4	
	p^a	0.61	0.12	0.31	0.25	0.41	
Home DBP in the evening in participants with normal-high BP (mmHg)	Placebo	72.1±8.8	2.7±6.6	4.1±5.9	6.6±6.7	4.2±7.2	0.90
	Eggplant	75.0±8.6	-0.6±4.9	0.6±5.2	3.8±4.6	3.1±8.1	
	p^a	0.23	0.044*	0.029*	0.083	0.93	
Home SBP in the evening in participants with grade 1 hypertension (mmHg)	Placebo	133.7±10.7	-4.3±6.8	4.2±9.3	3.1±11.3	3.7±9.9	0.70
	Eggplant	138.7±9.7	-1.0±8.9	3.4±7.9	3.4±13.5	1.9±13.5	
	p^a	0.27	0.31	0.82	0.96	0.64	
Home DBP in the evening in participants with grade 1 hypertension (mmHg)	Placebo	82.2±7.9	-3.6±5.2	0.8±5.7	2.8±6.2	1.8±6.1	0.54
	Eggplant	83.6±7.0	-0.2±5.0	2.2±4.3	2.7±8.1	2.5±3.6	
	p^a	0.69	0.13	0.52	0.97	0.75	

Values are shown as mean ± standard deviation. p^a: Student's *t*-test was performed. p^b: repeated-measure analysis of variance was performed. *$p < 0.05$, **$p < 0.01$ vs. placebo group.

ΔWeek 4: changes in values from baseline to week 4; ΔWeek 8: changes in values from baseline to week 8; ΔWeek 12: changes in values from baseline to week 12; ΔWeek 16: changes in values from baseline to four weeks after the end of ingestion. BP: blood pressure; SBP: systolic blood pressure; DBP: diastolic blood pressure.

Table S2. Blood pressure (measured value)

		Week 0	Week 4	Week 8	Week 12	Week 16	*time x food interaction, p^d*
Hospital SBP (mmHg)	Placebo	134.1±14.0	131.4±12.5	133.2±12.3	135.1±12.2	132.2±14.0	0.018*
	Eggplant	131.8±10.6	132.2±14.6	128.9±12.0	130.0±9.6	131.1±12.5	
	p^a	0.43	0.79	0.13	0.046*	0.70	
	p^b	-	0.12	0.63	0.58	0.36	
	p^c	-	0.87	0.089	0.24	0.72	
Hospital DBP (mmHg)	Placebo	83.5±10.4	83.8±9.0	85.5±8.0	84.7±9.7	84.4±10.0	0.13
	Eggplant	83.0±8.5	83.2±10.7	81.7±10.5	82.4±9.0	83.5±8.6	
	p^a	0.80	0.77	0.075	0.29	0.69	
	p^b	-	0.75	0.054	0.18	0.50	
	p^c	-	0.86	0.21	0.60	0.65	
Hospital SBP in participants with normal-high BP (mmHg)	Placebo	125.5±9.1	125.8±10.2	128.1±9.8	129.5±10.4	127.8±11.2	0.14
	Eggplant	127.1±6.8	127.9±13.4	125.0±9.8	127.6±9.1	128.8±11.3	
	p^a	0.48	0.54	0.27	0.47	0.74	
	p^b	-	0.89	0.29	0.11	0.34	
	p^c	-	0.77	0.30	0.76	0.45	
Hospital DBP in participants with normal-high BP (mmHg)	Placebo	77.0±5.6	79.5±6.3	81.8±5.6	79.3±6.6	80.3±8.3	0.043*
	Eggplant	80.1±6.4	80.6±9.9	79.3±9.7	81.0±8.4	82.0±8.3	
	p^a	0.069	0.62	0.25	0.43	0.44	
	p^b	-	0.033#	0.000021##	0.034#	0.031#	
	p^c	-	0.69	0.47	0.42	0.17	
Hospital SBP in participants with grade 1 hypertension (mmHg)	Placebo	149.0±5.9	140.9±10.2	142.1±11.2	144.7±8.5	140.0±15.4	0.028*
	Eggplant	146.1±5.8	145.1±10.0	140.4±10.9	137.1±7.4	138.0±14.3	
	p^a	0.26	0.34	0.73	0.037*	0.75	
	p^b	-	0.00014##	0.0053##	0.047#	0.011#	
	p^c	-	0.74	0.15	0.0044##	0.096	
Hospital DBP in participants with grade 1 hypertension (mmHg)	Placebo	94.8±5.9	91.4±8.0	92.1±7.5	94.1±6.6	91.5±8.9	0.079
	Eggplant	91.7±8.5	90.9±9.6	89.1±9.7	86.8±9.9	87.9±8.3	
	p^a	0.30	0.89	0.41	0.041*	0.34	
	p^b	-	0.045#	0.14	0.65	0.11	
	p^c	-	0.73	0.27	0.0081##	0.0095##	
Home SBP in the morning (mmHg)	Placebo	131.5±12.7	131.1±11.5	133.4±12.8	135.2±11.5	132.8±13.6	0.54
	Eggplant	133.6±14.1	128.7±12.0	132.6±11.5	134.7±14.8	134.9±13.3	
	p^a	0.49	0.38	0.78	0.87	0.50	
	p^b	-	0.78	0.11	0.0084##	0.28	
	p^c	-	0.0015##	0.50	0.42	0.42	
Home DBP in the morning (mmHg)	Placebo	81.2±10.6	81.7±9.3	83.0±10.4	83.8±9.6	83.1±10.3	0.54
	Eggplant	83.1±9.8	80.7±9.5	82.6±10.1	84.1±9.9	83.8±9.9	
	p^a	0.44	0.67	0.89	0.90	0.76	
	p^b	-	0.63	0.058	0.0098##	0.032#	
	p^c	-	0.014#	0.62	0.21	0.47	
Home SBP in the morning in	Placebo	127.1±11.0	127.7±10.3	129.9±11.7	131.6±9.7	128.0±11.5	0.54
	Eggplant	129.6±12.1	126.4±12.1	130.5±11.8	131.9±14.1	133.2±14.0	

participants with normal-high BP (mmHg)	p^a	0.44	0.67	0.85	0.91	0.15	
	p^b	-	0.65	0.040[#]	0.0020[##]	0.55	
	p^c	-	0.017[#]	0.52	0.12	0.038[#]	
Home DBP in the morning in participants with normal-high BP (mmHg)	Placebo	77.3±9.4	79.1±9.0	79.3±9.1	80.6±8.2	79.6±9.2	0.31
	Eggplant	81.3±8.4	79.5±9.6	81.3±10.2	82.6±9.9	82.7±10.4	
	p^a	0.12	0.87	0.45	0.44	0.25	
	p^b	-	0.10	0.12	0.0083[##]	0.086	
	p^c	-	0.033[#]	0.98	0.11	0.17	
Home SBP in the morning in participants with grade 1 hypertension (mmHg)	Placebo	139.0±12.2	137.0±11.6	139.5±12.7	141.5±12.1	141.2±13.0	0.81
	Eggplant	145.4±13.7	135.8±8.5	139.0±7.8	143.0±14.3	140.0±9.8	
	p^a	0.24	0.79	0.91	0.79	0.81	
	p^b	-	0.42	0.83	0.41	0.36	
	p^c	-	0.038[#]	0.067	0.51	0.19	
Home DBP in the morning in participants with grade 1 hypertension (mmHg)	Placebo	88.0±9.2	86.2±8.3	89.3±9.5	89.2±9.5	89.2±9.3	0.70
	Eggplant	88.4±12.2	84.6±8.7	86.6±9.0	88.6±8.9	87.1±8.0	
	p^a	0.91	0.66	0.50	0.87	0.58	
	p^b	-	0.26	0.32	0.44	0.16	
	p^c	-	0.20	0.33	0.95	0.66	
Home SBP in the evening (mmHg)	Placebo	126.7±11.9	126.7±10.4	130.7±12.7	133.0±12.6	132.5±13.3	0.88
	Eggplant	127.8±12.1	127.0±13.2	129.9±13.7	132.6±15.5	131.6±14.3	
	p^a	0.69	0.92	0.80	0.89	0.78	
	p^b	-	0.99	0.0044[##]	0.00082[##]	0.0021[##]	
	p^c	-	0.47	0.093	0.0032[##]	0.030[#]	
Home DBP in the evening (mmHg)	Placebo	75.8±9.7	76.2±8.0	78.7±8.3	81.0±9.6	79.1±9.8	0.76
	Eggplant	77.1±8.9	76.6±10.3	78.1±11.1	80.7±11.2	80.1±11.4	
	p^a	0.54	0.84	0.81	0.88	0.68	
	p^b	-	0.71	0.0040[##]	0.000012[##]	0.0038[##]	
	p^c	-	0.56	0.24	0.00054[##]	0.018[#]	
Home SBP in the evening in participants with normal-high BP (mmHg)	Placebo	122.7±10.8	125.1±9.1	126.5±10.5	130.8±10.5	129.6±13.2	0.86
	Eggplant	124.2±10.6	123.4±12.6	125.9±11.5	129.4±13.4	128.5±13.2	
	p^a	0.61	0.56	0.83	0.67	0.78	
	p^b	-	0.15	0.023[#]	0.00077[##]	0.0065[##]	
	p^c	-	0.52	0.24	0.00090[##]	0.039[#]	
Home DBP in the evening in participants with normal-high BP (mmHg)	Placebo	72.1±8.8	74.8±7.1	76.2±7.9	78.7±8.4	76.3±8.9	0.90
	Eggplant	75.0±8.6	74.4±10.2	75.6±10.5	78.8±10.6	78.1±11.5	
	p^a	0.23	0.87	0.82	0.97	0.52	
	p^b	-	0.047[#]	0.0017[##]	0.000035[##]	0.0068[##]	
	p^c	-	0.55	0.55	0.00021[##]	0.054	
Home SBP in the evening in participants with grade 1 hypertension (mmHg)	Placebo	133.7±10.7	129.4±12.3	138.0±13.3	136.8±15.1	137.5±12.3	0.70
	Eggplant	138.7±9.7	137.8±8.1	142.1±13.1	142.1±18.1	140.6±14.0	
	p^a	0.27	0.085	0.46	0.45	0.57	
	p^b	-	0.028[#]	0.10	0.31	0.17	
	p^c	-	0.75	0.24	0.47	0.53	
Home DBP in the evening in	Placebo	82.2±7.9	78.6±9.2	83.0±7.5	85.1±10.6	84.0±9.4	0.54
	Eggplant	83.6±7.0	83.4±7.6	85.7±9.7	86.3±11.9	86.0±8.9	

participants with grade 1 hypertension (mmHg)	p^a	0.69	0.21	0.45	0.80	0.61
	p^b	-	0.017#	0.62	0.099	0.29
	p^c	-	0.91	0.17	0.35	0.073

Values are shown as mean ± standard deviation. $p^{a–c}$: Student's *t*-test was performed. p^d: repeated-measure analysis of variance was performed. *$p < 0.05$, **$p < 0.01$ vs. placebo group. #$p < 0.05$, ##$p < 0.01$ vs. week 0. BP: blood pressure; SBP: systolic blood pressure; DBP: diastolic blood pressure.

Table S3. VAS scores indicating stress and POMS-2 scores indicating psychological state (measured value)

		Week 0	Week 4	Week 8	Week 12	Week 16	*time x food interaction, p^d*
Stress before UKT (mm)	Placebo	50.6±18.7	52.0±21.3	57.0±19.0	54.8±20.6	54.2±20.7	
	Eggplant	54.7±17.1	57.9±16.7	57.5±16.6	55.3±17.3	54.9±17.8	
	p^a	0.32	0.18	0.92	0.92	0.86	0.30
	p^b	-	0.57	0.012[#]	0.13	0.19	
	p^c	-	0.39	0.46	0.88	0.95	
Stress after UKT (mm)	Placebo	32.9±22.4	33.1±20.6	35.1±20.8	39.4±22.6	33.4±23.0	
	Eggplant	28.8±17.0	33.6±18.2	33.2±17.6	34.8±20.7	35.4±19.2	
	p^a	0.36	0.92	0.66	0.36	0.69	0.47
	p^b	-	0.93	0.37	0.061	0.87	
	p^c	-	0.18	0.19	0.13	0.091	
Stress before UKT in participants with normal-high BP (mm)	Placebo	52.4±17.1	51.8±18.6	55.8±15.8	56.3±17.7	53.2±18.5	
	Eggplant	56.6±18.7	60.2±17.5	59.4±17.0	56.6±18.3	55.6±18.4	
	p^a	0.40	0.097	0.43	0.95	0.65	0.27
	p^b	-	0.86	0.22	0.27	0.81	
	p^c	-	0.44	0.53	0.99	0.82	
Stress after UKT in participants with normal-high BP (mm)	Placebo	37.6±22.5	32.5±19.7	33.2±19.9	38.5±22.1	34.2±23.5	
	Eggplant	29.9±18.1	32.7±19.5	32.8±18.2	34.9±21.7	34.4±18.6	
	p^a	0.18	0.96	0.94	0.56	0.97	0.73
	p^b	-	0.10	0.069	0.82	0.44	
	p^c	-	0.53	0.49	0.32	0.34	
Stress before UKT in participants with grade 1 hypertension (mm)	Placebo	47.4±21.4	52.3±25.9	59.1±24.1	52.3±25.4	55.8±24.6	
	Eggplant	49.1±9.5	51.0±11.9	51.7±15.0	51.2±14.3	53.1±16.6	
	p^a	0.79	0.87	0.41	0.91	0.78	0.62
	p^b	-	0.19	0.023[#]	0.31	0.051	
	p^c	-	0.74	0.71	0.76	0.60	
Stress after UKT in participants with grade 1 hypertension (mm)	Placebo	24.9±20.6	34.3±22.8	38.5±22.6	40.9±24.2	32.1±22.9	
	Eggplant	25.3±13.5	36.1±14.3	34.3±16.4	34.4±18.5	38.3±21.8	
	p^a	0.95	0.83	0.64	0.50	0.52	0.46
	p^b	-	0.018[#]	0.0025[##]	0.0072[##]	0.041[#]	
	p^c	-	0.038[#]	0.094	0.11	0.072	
Anger-Hostility	Placebo	9.0±7.0	8.6±6.4	9.0±7.2	8.3±7.2	8.0±6.1	
	Eggplant	7.6±6.2	7.2±6.5	7.3±7.3	5.8±5.6	6.9±7.8	
	p^a	0.36	0.33	0.32	0.085	0.52	0.51
	p^b	-	0.71	0.98	0.49	0.27	
	p^c	-	0.56	0.72	0.041[#]	0.48	
Anger-Hostility in participants with normal-high BP	Placebo	9.2±7.1	8.9±6.2	9.5±7.0	9.6±7.3	8.6±5.9	
	Eggplant	7.7±6.7	7.2±6.7	7.4±7.5	5.8±5.6	7.0±8.3	
	p^a	0.41	0.34	0.29	0.040*	0.42	0.23
	p^b	-	0.79	0.81	0.79	0.63	
	p^c	-	0.50	0.66	0.072	0.48	

Anger-Hostility in participants with grade 1 hypertension	Placebo	8.5±7.2	8.1±6.8	8.1±7.6	6.2±6.6	6.9±6.5	0.85
	Eggplant	7.2±4.5	7.1±6.4	7.1±7.4	5.9±5.8	6.8±6.5	
	p^a	0.65	0.72	0.77	0.91	0.98	
	p^b	-	0.80	0.67	0.0103[#]	0.12	
	p^c	-	0.95	0.96	0.37	0.83	
Confusion-Bewilderment	Placebo	13.2±4.3	12.3±4.5	12.3±4.9	12.3±5.0	12.5±5.7	0.068
	Eggplant	12.3±4.6	10.8±4.8	11.6±4.6	9.9±4.1	11.4±4.8	
	p^a	0.37	0.14	0.56	0.026	0.37	
	p^b	-	0.10	0.072	0.11	0.22	
	p^c	-	0.020[#]	0.35	0.00080[##]	0.15	
Confusion-Bewilderment in participants with normal-high BP	Placebo	13.7±4.0	12.8±3.9	12.9±4.5	13.4±4.5	13.8±5.4	0.18
	Eggplant	12.1±4.7	10.9±5.0	11.8±4.6	10.5±4.3	12.0±5.1	
	p^a	0.19	0.11	0.40	0.021*	0.22	
	p^b	-	0.19	0.19	0.68	0.96	
	p^c	-	0.063	0.65	0.017[#]	0.76	
Confusion-Bewilderment in participants with grade 1 hypertension	Placebo	12.4±4.8	11.4±5.6	11.2±5.4	10.4±5.3	10.3±5.6	0.15
	Eggplant	12.9±4.7	10.4±4.3	11.1±4.7	8.2±3.0	9.7±3.6	
	p^a	0.81	0.66	0.97	0.21	0.78	
	p^b	-	0.35	0.24	0.0059[##]	0.0053[##]	
	p^c	-	0.19	0.41	0.019[#]	0.088	
Depression-Dejection	Placebo	9.5±8.3	9.0±7.7	9.0±8.0	8.0±7.3	7.8±7.7	0.84
	Eggplant	7.2±6.3	6.7±7.1	6.9±6.3	5.4±5.5	6.3±7.0	
	p^a	0.18	0.17	0.22	0.085	0.38	
	p^b	-	0.56	0.55	0.18	0.17	
	p^c	-	0.53	0.75	0.042[#]	0.21	
Depression-Dejection in participants with normal-high BP	Placebo	9.5±8.7	9.4±7.2	9.8±7.3	8.4±6.5	8.2±6.7	0.94
	Eggplant	7.5±6.9	5.9±6.4	6.2±5.6	5.2±5.5	6.7±7.4	
	p^a	0.35	0.067	0.048*	0.061	0.43	
	p^b	-	0.91	0.79	0.48	0.46	
	p^c	-	0.022[#]	0.080	0.041[#]	0.36	
Depression-Dejection in participants with grade 1 hypertension	Placebo	9.5±7.8	8.3±8.8	7.5±9.2	7.3±8.8	7.0±9.4	0.18
	Eggplant	6.4±4.1	9.0±8.9	9.2±7.9	5.8±6.0	5.1±5.7	
	p^a	0.30	0.86	0.64	0.66	0.59	
	p^b	-	0.45	0.19	0.14	0.17	
	p^c	-	0.39	0.30	0.69	0.38	
Fatigue-Inertia	Placebo	7.5±5.1	7.1±4.4	6.9±5.0	6.1±4.2	6.6±5.2	0.80
	Eggplant	7.2±4.1	7.1±4.5	6.8±3.8	5.7±4.0	6.8±4.3	
	p^a	0.80	0.99	0.92	0.63	0.84	
	p^b	-	0.57	0.15	0.022[#]	0.052	
	p^c	-	0.94	0.60	0.025[#]	0.52	
Fatigue-Inertia in participants with normal-high BP	Placebo	8.3±5.4	8.1±4.0	8.3±4.8	7.3±4.0	8.1±5.4	0.75
	Eggplant	7.1±4.2	7.0±4.4	6.6±3.7	5.7±4.4	6.9±4.6	
	p^a	0.37	0.36	0.17	0.17	0.39	
	p^b	-	0.80	1.0	0.24	0.73	

	p^c	-	0.90	0.47	0.039#	0.76	
Fatigue-Inertia in participants with grade 1 hypertension	Placebo	6.1±4.5	5.5±4.8	4.5±4.5	4.1±3.8	3.9±3.5	0.61
	Eggplant	7.6±3.8	7.6±4.8	7.3±4.1	5.6±2.9	6.4±3.3	
	p^a	0.42	0.33	0.14	0.32	0.098	
	p^b	-	0.51	0.015#	0.012#	0.0079##	
	p^c	-	1.00	0.93	0.32	0.58	
Tension-Anxiety	Placebo	14.7±8.0	13.6±6.6	13.4±6.5	13.2±7.5	13.2±6.9	0.36
	Eggplant	13.4±5.4	11.4±5.1	12.7±5.0	11.1±4.3	12.4±6.2	
	p^a	0.40	0.12	0.62	0.13	0.62	
	p^b	-	0.087	0.11	0.18	0.10	
	p^c	-	0.038#	0.52	0.035#	0.42	
Tension-Anxiety in participants with normal-high BP	Placebo	15.3±8.6	14.1±7.0	14.0±6.3	13.9±7.8	13.6±6.6	0.40
	Eggplant	13.1±5.4	11.0±5.2	12.9±5.1	11.1±4.5	12.5±7.0	
	p^a	0.26	0.077	0.47	0.13	0.56	
	p^b	-	0.19	0.20	0.36	0.19	
	p^c	-	0.0093##	0.84	0.075	0.69	
Tension-Anxiety in participants with grade 1 hypertension	Placebo	13.7±7.0	12.7±5.8	12.3±6.9	12.1±7.0	12.5±7.4	0.79
	Eggplant	14.4±5.7	12.7±4.7	12.2±5.3	11.0±3.6	12.2±3.0	
	p^a	0.80	1.00	0.99	0.61	0.91	
	p^b	-	0.27	0.36	0.26	0.34	
	p^c	-	0.58	0.52	0.27	0.39	
Vigor-Activity	Placebo	12.7±6.5	11.9±5.7	12.4±6.5	12.4±6.3	11.9±6.7	0.41
	Eggplant	13.1±7.1	13.4±7.3	12.9±7.4	12.6±7.5	12.3±7.3	
	p^a	0.82	0.31	0.75	0.89	0.82	
	p^b	-	0.19	0.54	0.70	0.33	
	p^c	-	0.69	0.85	0.55	0.28	
Vigor-Activity in participants with normal-high BP	Placebo	11.8±5.1	11.3±5.4	11.9±5.7	11.8±5.3	11.4±6.1	0.60
	Eggplant	13.9±6.8	13.3±7.6	12.7±7.2	12.7±8.0	12.8±7.4	
	p^a	0.22	0.28	0.66	0.61	0.47	
	p^b	-	0.60	0.91	0.97	0.74	
	p^c	-	0.41	0.12	0.19	0.23	
Vigor-Activity in participants with grade 1 hypertension	Placebo	14.3±8.3	12.7±6.4	13.3±7.9	13.5±7.7	12.7±7.7	0.54
	Eggplant	10.7±7.8	13.6±6.7	13.7±8.5	12.3±6.3	10.8±7.5	
	p^a	0.30	0.77	0.92	0.70	0.55	
	p^b	-	0.12	0.18	0.58	0.21	
	p^c	-	0.22	0.24	0.24	0.93	
Friendliness	Placebo	10.7±4.1	10.8±3.2	11.2±3.7	10.3±3.5	9.6±3.2	0.54
	Eggplant	10.6±4.4	10.7±4.1	10.5±4.4	10.1±3.8	9.9±4.3	
	p^a	0.98	0.92	0.44	0.81	0.75	
	p^b	-	0.78	0.28	0.56	0.063	
	p^c	-	0.86	0.84	0.17	0.080	
Friendliness in participants with normal-high BP	Placebo	10.2±4.0	10.8±3.5	11.7±3.4	10.4±3.4	9.6±3.5	0.16
	Eggplant	11.3±4.1	10.9±4.2	10.5±4.6	10.3±3.6	10.3±4.0	
	p^a	0.36	1.00	0.29	0.90	0.49	

	p^b	-	0.39	0.039#	0.85	0.43	
	p^c	-	0.40	0.23	0.029#	0.096	
Friendliness in participants with grade 1 hypertension	Placebo	11.4±4.3	10.7±2.8	10.4±4.0	10.2±3.7	9.6±2.8	0.83
	Eggplant	8.8±4.9	10.3±4.0	10.6±3.8	9.7±4.6	8.6±5.2	
	p^a	0.19	0.78	0.93	0.76	0.52	
	p^b	-	0.34	0.15	0.12	0.019#	
	p^c	-	0.18	0.15	0.15	0.62	
TMD score before UKT	Placebo	41.1±30.1	38.8±26.5	38.1±29.1	35.6±27.8	36.1±29.4	0.46
	Eggplant	34.6±24.2	29.8±27.0	32.5±23.3	25.3±22.4	31.6±27.7	
	p^a	0.30	0.15	0.36	0.079	0.49	
	p^b	-	0.35	0.19	0.13	0.13	
	p^c	-	0.14	0.49	0.011#	0.37	
TMD score before UKT in participants with normal-high BP	Placebo	44.2±30.7	41.9±24.7	42.6±27.6	40.8±26.2	40.9±28.6	0.66
	Eggplant	33.6±26.2	28.6±27.3	32.2±22.0	25.6±23.5	32.3±29.9	
	p^a	0.18	0.069	0.14	0.031*	0.29	
	p^b	-	0.50	0.60	0.52	0.50	
	p^c	-	0.11	0.62	0.052	0.74	
TMD score before UKT in participants with grade 1 hypertension	Placebo	35.8±29.2	33.3±29.4	30.2±30.7	26.5±28.9	27.8±30.0	0.50
	Eggplant	37.9±17.9	33.2±27.6	33.3±28.4	24.1±20.2	29.4±21.3	
	p^a	0.85	0.99	0.81	0.83	0.89	
	p^b	-	0.52	0.14	0.012#	0.027#	
	p^c	-	0.64	0.65	0.13	0.28	

Values are shown as mean ± standard deviation. $p^{a–c}$: Student's *t*-test was performed. p^d: repeated-measure analysis of variance was performed. *$p < 0.05$, **$p < 0.01$ vs. placebo group. #$p < 0.05$, ##$p < 0.01$ vs. week 0. BP: Blood pressure; TMD: Total Mood Disturbance; UKT: Uchida-Kraepelin Psychodiagnostic Test.

Table S4. Changes in VAS and POMS-2 scores from week 0

		Week 0	ΔWeek 4	ΔWeek 8	ΔWeek 12	ΔWeek 16	*time x food interaction, p*[b]
Stress before UKT (mm)	Placebo	50.6±18.7	1.4±15.9	6.5±15.7	4.2±17.4	3.6±17.1	0.30
	Eggplant	54.7±17.1	3.2±22.4	2.8±22.3	0.6±21.7	0.3±22.3	
	p[a]	0.32	0.69	0.40	0.41	0.46	
Stress after UKT (mm)	Placebo	32.9±22.4	0.2±16.1	2.2±15.4	6.4±21.4	0.5±19.5	0.47
	Eggplant	28.8±17.0	4.8±21.1	4.4±19.7	6.0±23.1	6.6±22.9	
	p[a]	0.36	0.28	0.58	0.94	0.21	
Stress before UKT in participants with normal-high BP (mm)	Placebo	52.4±17.1	-0.6±16.9	3.5±13.9	3.9±17.5	0.8±17.9	0.27
	Eggplant	56.6±18.7	3.7±24.3	2.9±23.4	0.0±22.5	-1.0±22.6	
	p[a]	0.40	0.47	0.91	0.49	0.74	
Stress after UKT in participants with normal-high BP (mm)	Placebo	37.6±22.5	-5.1±15.2	-4.4±11.8	0.9±20.6	-3.3±21.9	0.73
	Eggplant	29.9±18.1	2.8±23.1	2.9±21.2	5.0±25.4	4.5±24.1	
	p[a]	0.18	0.15	0.13	0.52	0.22	
Stress before UKT in participants with grade 1 hypertension (mm)	Placebo	47.4±21.4	4.9±13.9	11.7±17.8	4.9±17.7	8.4±15.2	0.62
	Eggplant	49.1±9.5	1.9±16.6	2.6±19.9	2.1±20.1	-4.0±22.2	
	p[a]	0.79	0.63	0.25	0.73	0.57	
Stress after UKT in participants with grade 1 hypertension (mm)	Placebo	24.9±20.6	9.4±13.7	13.6±14.3	16.0±19.7	7.2±12.4	0.46
	Eggplant	25.3±13.5	10.8±13.0	9.0±14.2	9.1±15.1	13.0±18.9	
	p[a]	0.95	0.81	0.45	0.38	0.37	
Anger-Hostility	Placebo	9.0±7.0	-0.3±5.9	0.0±4.9	-0.6±5.6	-1.0±5.6	0.51
	Eggplant	7.6±6.2	-0.4±4.0	-0.3±4.1	-1.8±4.9	-0.6±5.1	
	p[a]	0.36	0.97	0.79	0.35	0.77	
Anger-Hostility in participants with normal-high BP	Placebo	9.2±7.1	-0.3±6.5	0.3±5.6	0.3±6.6	-0.6±6.4	0.23
	Eggplant	7.7±6.7	-0.5±3.7	-0.3±3.5	-1.9±5.2	-0.7±4.8	
	p[a]	0.41	0.93	0.66	0.18	0.97	
Anger-Hostility in participants with grade 1 hypertension	Placebo	8.5±7.2	-0.3±5.0	-0.4±3.5	-2.3±3.0	-1.6±3.7	0.85
	Eggplant	7.2±4.5	-0.1±5.1	-0.1±6.1	-1.3±4.2	-0.4±6.0	
	p[a]	0.65	0.92	0.88	0.53	0.57	
Confusion-Bewilderment	Placebo	13.2±4.3	-0.9±3.5	-1.0±3.4	-1.0±3.7	-0.8±3.9	0.068
	Eggplant	12.3±4.6	-1.6±3.9	-0.7±4.4	-2.4±4.0	-0.9±3.8	
	p[a]	0.37	0.44	0.75	0.097	0.83	
Confusion-Bewilderment in participants with normal-high BP	Placebo	13.7±4.0	-0.9±3.3	-0.8±3.2	-0.3±4.2	0.0±4.3	0.18
	Eggplant	12.1±4.7	-1.3±3.5	-0.3±3.7	-1.7±3.4	-0.2±3.1	
	p[a]	0.19	0.66	0.60	0.21	0.83	
Confusion-Bewilderment in participants with grade 1 hypertension	Placebo	12.4±4.8	-1.0±4.0	-1.2±3.7	-2.0±2.4	-2.1±2.5	0.15
	Eggplant	12.9±4.7	-2.4±5.2	-1.8±6.1	-4.7±4.8	-3.2±5.0	
	p[a]	0.81	0.45	0.78	0.081	0.55	
Depression-Dejection	Placebo	9.5±8.3	-0.5±5.3	-0.5±5.7	-1.5±7.1	-1.7±7.9	0.84
	Eggplant	7.2±6.3	-0.6±5.3	-0.3±5.1	-1.9±5.3	-0.9±4.5	
	p[a]	0.18	0.96	0.84	0.81	0.61	

Depression-Dejection in participants with normal-high BP	Placebo	9.5±8.7	-0.1±5.2	0.3±5.7	-1.1±8.0	-1.3±8.6	0.94
	Eggplant	7.5±6.9	-1.6±3.4	-1.3±3.7	-2.3±5.5	-0.8±4.6	
	p[a]	0.35	0.23	0.23	0.54	0.81	
Depression-Dejection in participants with grade 1 hypertension	Placebo	9.5±7.8	-1.1±5.6	-2.0±5.6	-2.2±5.4	-2.5±6.6	0.18
	Eggplant	6.4±4.1	2.6±8.4	2.8±7.5	-0.7±4.9	-1.3±4.3	
	p[a]	0.30	0.21	0.089	0.49	0.65	
Fatigue-Inertia	Placebo	7.5±5.1	-0.3±3.5	-0.6±2.5	-1.3±3.5	-0.9±2.9	0.80
	Eggplant	7.2±4.1	-0.1±4.5	-0.4±4.4	-1.5±3.9	-0.4±3.8	
	p[a]	0.80	0.78	0.84	0.83	0.53	
Fatigue-Inertia in participants with normal-high BP	Placebo	8.3±5.4	-0.2±3.8	0.0±2.5	-0.9±3.9	-0.2±2.8	0.75
	Eggplant	7.1±4.2	-0.1±3.1	-0.4±3.2	-1.3±3.2	-0.2±3.1	
	p[a]	0.37	0.90	0.57	0.68	0.99	
Fatigue-Inertia in participants with grade 1 hypertension	Placebo	6.1±4.5	-0.5±3.1	-1.5±2.1	-2.0±2.7	-2.1±2.7	0.61
	Eggplant	7.6±3.8	0.0±7.7	-0.2±7.3	-2.0±5.6	-1.1±5.8	
	p[a]	0.42	0.85	0.61	1.00	0.56	
Tension-Anxiety	Placebo	14.7±8.0	-1.2±4.3	-1.3±5.3	-1.5±6.9	-1.5±5.9	0.36
	Eggplant	13.4±5.4	-2.0±5.5	-0.7±6.3	-2.3±6.3	-1.0±7.1	
	p[a]	0.40	0.47	0.63	0.59	0.70	
Tension-Anxiety in participants with normal-high BP	Placebo	15.3±8.6	-1.2±4.7	-1.3±5.0	-1.4±7.8	-1.7±6.4	0.40
	Eggplant	13.1±5.4	-2.0±3.8	-0.2±4.8	-1.9±5.4	-0.6±7.1	
	p[a]	0.26	0.49	0.42	0.79	0.54	
Tension-Anxiety in participants with grade 1 hypertension	Placebo	13.7±7.0	-1.1±3.6	-1.5±6.0	-1.6±5.3	-1.3±4.9	0.79
	Eggplant	14.4±5.7	-1.8±9.3	-2.2±9.9	-3.4±8.8	-2.2±7.4	
	p[a]	0.80	0.79	0.82	0.53	0.71	
Vigor-Activity	Placebo	12.7±6.5	-0.9±4.2	-0.3±3.3	-0.3±5.3	-0.8±5.4	0.41
	Eggplant	13.1±7.1	0.3±4.5	-0.2±5.2	-0.4±4.4	-0.8±4.4	
	p[a]	0.82	0.24	0.88	0.91	0.98	
Vigor-Activity in participants with normal-high BP	Placebo	11.8±5.1	-0.5±4.5	0.1±3.6	0.0±5.3	-0.4±5.8	0.60
	Eggplant	13.9±6.8	-0.6±3.4	-1.2±4.0	-1.1±4.4	-1.1±4.7	
	p[a]	0.22	0.93	0.22	0.41	0.62	
Vigor-Activity in participants with grade 1 hypertension	Placebo	14.3±8.3	-1.6±3.8	-1.0±2.8	-0.8±5.4	-1.6±4.7	0.54
	Eggplant	10.7±7.8	2.9±6.5	3.0±7.1	1.7±3.9	0.1±3.6	
	p[a]	0.30	0.042*	0.14	0.25	0.36	
Friendliness	Placebo	10.7±4.1	0.1±3.3	0.6±3.4	-0.3±3.7	-1.0±3.5	0.54
	Eggplant	10.6±4.4	0.1±2.8	-0.1±3.3	-0.5±2.3	-0.8±2.5	
	p[a]	0.98	0.93	0.37	0.79	0.67	
Friendliness in participants with normal-high BP	Placebo	10.2±4.0	0.6±3.6	1.5±3.5	0.2±4.1	-0.6±3.9	0.16
	Eggplant	11.3±4.1	-0.4±2.5	-0.7±3.1	-1.0±2.3	-0.9±2.8	
	p[a]	0.36	0.23	0.017*	0.21	0.74	
Friendliness in participants with grade 1 hypertension	Placebo	11.4±4.3	-0.7±2.6	-1.0±2.6	-1.2±2.8	-1.8±2.6	0.83
	Eggplant	8.8±4.9	1.6±3.2	1.8±3.4	0.9±1.7	-0.2±1.3	
	p[a]	0.19	0.077	0.033*	0.057	0.11	
TMD score before UKT	Placebo	41.1±30.1	-2.4±16.2	-3.1±14.7	-5.6±22.8	-5.0±21.0	0.46
	Eggplant	34.6±24.2	-4.9±19.3	-2.1±18.3	-9.4±21.1	-3.1±20.6	

	p^a	0.30	0.54	0.81	0.45	0.68	
TMD score before UKT in participants with normal-high BP	Placebo	44.2±30.7	-2.3±17.3	-1.6±15.4	-3.4±27.0	-3.3±24.7	0.66
	Eggplant	33.6±26.2	-4.9±15.5	-1.3±13.7	-7.9±20.2	-1.3±20.1	
	p^a	0.18	0.57	0.94	0.49	0.74	
TMD score before UKT in participants with grade 1 hypertension	Placebo	35.8±29.2	-2.5±14.5	-5.6±13.7	-9.3±12.5	-8.0±12.5	0.50
	Eggplant	37.9±17.9	-4.7±29.0	-4.6±29.2	-13.8±24.3	-8.4±22.1	
	p^a	0.85	0.81	0.91	0.55	0.95	

Values are shown as mean ± standard deviation. p^a: Student's *t*-test was used for data analysis. $*p < 0.05$, $**p < 0.01$ vs. placebo group. ΔWeek 4: changes in values from baseline to week 4; ΔWeek 8: changes in values from baseline to week 8; ΔWeek 12: changes in values from baseline to week 12; ΔWeek 16: changes in values from baseline to four weeks after the end of ingestion. BP: Blood pressure; TMD: Total Mood Disturbance; UKT: Uchida-Kraepelin Psychodiagnostic Test.

Table S5. Dietary nutrients consumed by participants during the study.

		Week 0	ΔWeek 4	ΔWeek 8	ΔWeek 12	ΔWeek 16
Calorie (kcal)	Placebo	1889.6±391.3	-19.7±253.2	-39.4±203.4	-5.9±252.4	-41.0±239.2
	Eggplant	1818.5±464.1	-18.7±301.0	-33.3±325.4	-88.7±326.8	-23.9±340.7
	p^a	0.47	0.99	0.92	0.22	0.80
Protein (g)	Placebo	64.7±16.2	0.4±9.0	0.1±10.0	0.8±10.6	-0.2±8.7
	Eggplant	64.9±17.7	-0.8±13.2	-0.5±10.0	-3.1±11.6	-0.8±14.1
	p^a	0.96	0.66	0.81	0.13	0.82
Lipid (g)	Placebo	64.7±20.7	0.1±9.6	-1.2±11.3	1.5±10.8	-0.4±11.2
	Eggplant	63.4±20.0	-2.4±12.2	-3.2±12.7	-5.7±12.9	-2.1±14.2
	p^a	0.79	0.33	0.46	0.009**	0.56
Carbohydrate (g)	Placebo	250.8±49.4	-5.5±41.3	-7.2±28.0	-5.4±36.8	-8.9±35.1
	Eggplant	234.4±65.2	2.1±43.5	0.3±47.2	-4.2±46.2	1.7±44.4
	p^a	0.21	0.43	0.39	0.90	0.25
Dietary fiber (g)	Placebo	12.4±4.0	0.2±2.0	0.4±2.2	0.4±2.0	0.1±2.0
	Eggplant	12.1±4.3	0.0±2.3	-0.4±2.4	-0.7±2.7	-0.3±2.9
	p^a	0.76	0.57	0.16	0.035*	0.57
Sodium chloride (g)	Placebo	9.3±3.0	-0.3±2.5	-0.5±1.9	-0.6±2.1	-0.6±2.0
	Eggplant	8.2±2.6	-0.1±1.9	0.0±1.6	-0.3±1.8	0.2±2.6
	p^a	0.091	0.62	0.23	0.49	0.13

Values are shown as mean ± standard deviation. p^a: Student's *t*-test was used for data analysis. *$p < 0.05$, **$p < 0.01$ vs. placebo group. ΔWeek 4: changes in values from baseline to week 4; ΔWeek 8: changes in values from baseline to week 8; ΔWeek 12: changes in values from baseline to week 12; ΔWeek 16: changes in values from baseline to four weeks after the end of ingestion.

Table S6. Body composition, complete blood count, liver function, renal function, lipid profiles, blood glucose profiles, and urinary tests.

		Week 0	ΔWeek 4	ΔWeek 8	ΔWeek 12	ΔWeek 16
BW (kg)	Placebo	60.5±10.6	-0.1±0.9	-0.1±1.1	0.3±1.5	0.4±1.3
	Eggplant	59.7±11.4	0.0±0.8	-0.1±0.8	0.2±1.0	0.4±1.1
	p^a	0.75	0.86	0.93	0.61	0.99
BFR (%)	Placebo	28.3±6.6	0.7±0.9	1.1±1.1	1.6±1.3	1.3±1.5
	Eggplant	27.7±6.7	1.0±0.9	1.3±1.0	1.4±1.1	1.4±1.0
	p^a	0.64	0.16	0.32	0.59	0.62
BMI (kg/m^2)	Placebo	22.9±2.7	0.0±0.3	0.0±0.4	0.1±0.6	0.2±0.5
	Eggplant	22.9±3.3	0.0±0.3	0.0±0.3	0.1±0.4	0.2±0.4
	p^a	0.90	0.99	0.94	0.52	0.95
WBC (×10^3/μL)	Placebo	5.3±1.0	0.1±0.9	-0.3±1.0	0.1±0.9	0.1±1.1
	Eggplant	5.9±1.3	0.2±0.8	-0.2±0.9	0.1±0.8	-0.1±0.9
	p^a	0.020*	0.81	0.62	0.96	0.39
RBC (×10^4/μL)	Placebo	473.4±38.8	7.8±19.8	12.2±21.9	9.2±22.9	4.5±20.4
	Eggplant	468.3±38.3	6.9±15.2	10.0±17.1	6.6±16.1	5.2±15.1
	p^a	0.52	0.81	0.61	0.54	0.86
Hb (g/dL)	Placebo	14.0±1.1	0.2±0.5	0.5±0.7	0.3±0.7	0.1±0.6
	Eggplant	14.0±1.2	0.2±0.4	0.4±0.5	0.3±0.6	0.2±0.5
	p^a	0.76	0.78	0.63	0.94	0.71
Htc (%)	Placebo	42.5±3.2	1.2±1.9	1.3±2.0	1.0±2.2	0.3±1.8
	Eggplant	42.4±3.3	0.8±1.5	1.1±1.7	0.8±1.6	0.2±1.4
	p^a	0.93	0.23	0.55	0.77	0.82
Plt (×10^4/μL)	Placebo	24.3±4.5	-0.5±2.0	-0.5±2.4	0.3±2.6	0.3±2.2
	Eggplant	24.7±5.4	0.8±1.9	0.3±2.8	1.0±2.6	1.4±2.9
	p^a	0.70	0.003**	0.12	0.24	0.053
AST (U/L)	Placebo	20.7±5.8	0.6±3.6	0.0±3.1	1.1±2.9	2.1±6.3
	Eggplant	20.5±4.0	0.0±2.5	0.6±3.7	1.0±3.1	2.0±4.1
	p^a	0.84	0.39	0.40	0.89	0.94
ALT (U/L)	Placebo	18.9±9.7	0.6±4.9	0.3±4.8	2.2±4.6	1.2±6.5
	Eggplant	18.7±5.2	0.9±5.5	0.7±6.3	2.0±5.0	2.5±8.4
	p^a	0.88	0.83	0.75	0.80	0.41
γ-GTP (U/L)	Placebo	27.0±23.1	1.2±5.8	1.2±6.6	3.5±12.8	0.7±15.6
	Eggplant	26.6±20.4	4.5±18.3	2.5±9.4	1.2±6.0	3.5±6.5
	p^a	0.93	0.25	0.45	0.28	0.27
ALP (U/L)	Placebo	205.0±54.5	5.3±13.8	9.2±18.2	13.7±23.3	13.8±46.4
	Eggplant	214.4±55.9	3.0±15.1	5.5±19.3	7.6±23.2	5.5±21.6
	p^a	0.40	0.44	0.36	0.23	0.29
LDH (U/L)	Placebo	183.1±28.4	-3.0±11.6	-8.6±16.8	-4.0±13.2	0.8±16.8
	Eggplant	193.2±27.3	-2.1±12.6	-6.9±12.0	-0.6±19.0	3.4±26.6
	p^a	0.079	0.72	0.58	0.33	0.58
BUN (mg/dL)	Placebo	14.3±3.6	-0.6±2.6	-0.2±3.1	-0.3±3.0	0.0±2.4
	Eggplant	14.6±3.9	-0.7±2.6	0.0±3.1	-1.0±3.0	-0.6±3.0

	p^a	0.68	0.84	0.74	0.28	0.26
CRE (mg/dL)	Placebo	0.8±0.2	0.0±0.0	0.0±0.1	0.0±0.1	0.0±0.0
	Eggplant	0.8±0.2	0.0±0.1	0.0±0.1	0.0±0.1	0.0±0.1
	p^a	0.79	0.89	0.85	0.33	0.57
UA (mg/dL)	Placebo	5.0±1.1	0.2±0.5	0.2±0.7	0.2±0.6	0.1±0.5
	Eggplant	5.2±1.3	0.2±0.5	0.1±0.5	0.1±0.5	0.0±0.6
	p^a	0.33	0.84	0.51	0.26	0.76
TC (mg/dL)	Placebo	223.6±31.7	6.0±16.3	5.8±17.0	10.5±15.9	1.9±17.4
	Eggplant	230.7±36.9	3.1±18.3	-2.0±19.8	4.8±20.9	-6.9±23.4
	p^a	0.32	0.41	0.048*	0.15	0.048*
LDL-C (mg/dL)	Placebo	136.2±28.4	3.3±14.4	6.2±15.7	7.8±13.7	3.4±15.5
	Eggplant	137.1±35.4	0.5±16.5	-0.2±16.5	4.1±18.8	-2.4±19.6
	p^a	0.89	0.38	0.063	0.30	0.13
HDL-C (mg/dL)	Placebo	67.3±16.4	-1.3±5.5	-1.0±6.7	2.1±5.7	1.6±6.5
	Eggplant	75.2±16.7	-2.2±5.8	-2.6±5.3	0.2±6.1	-1.4±6.4
	p^a	0.022*	0.41	0.22	0.13	0.036*
TG (mg/dL)	Placebo	100.5±40.9	0.3±26.9	2.0±26.8	1.0±30.8	-8.9±32.6
	Eggplant	97.1±44.3	11.0±34.5	9.4±34.5	0.8±37.3	-0.4±31.3
	p^a	0.70	0.10	0.26	0.98	0.22
FPG (mg/dL)	Placebo	90.7±10.2	-0.8±5.8	-1.7±5.0	-0.3±4.4	0.0±7.6
	Eggplant	89.0±8.8	-2.1±4.8	-1.7±3.7	-0.8±4.1	-1.0±6.2
	p^a	0.39	0.23	0.94	0.59	0.51
HbA1c (%)	Placebo	5.4±0.4	0.0±0.1	0.0±0.2	0.0±0.2	-0.1±0.2
	Eggplant	5.3±0.3	0.0±0.1	0.1±0.1	0.1±0.1	0.0±0.1
	p^a	0.19	0.35	0.25	0.20	0.12
Urine pH	Placebo	6.1±0.7	0.1±0.6	0.1±0.8	-0.2±0.8	0.0±0.8
	Eggplant	6.1±0.6	-0.1±0.7	-0.1±0.7	-0.1±0.7	-0.2±0.6
	p^a	0.85	0.14	0.40	0.79	0.28
Urine sugar	p^b	0.33	0.39	0.33	0.39	0.33
Urine protein	p^b	0.95	0.58	0.58	0.95	0.66
Urine occult blood	p^b	0.17	0.57	0.43	0.19	0.28
Urine urobilinogen	p^b	1.0	0.34	1.0	1.0	1.0
Urine ketones	p^b	1.0	1.0	1.0	1.0	1.0

Values are shown as mean ± standard deviation. p^a: Student's *t*-test was performed. *$p < 0.05$, **$p < 0.01$ vs. placebo group. p^b: Fisher's exact probability test was performed. ΔWeek 4: changes in values from baseline to week 4; ΔWeek 8: changes in values from baseline to week 8; ΔWeek 12: changes in values from baseline to week 12; ΔWeek 16: changes in values from baseline to four weeks after the end of ingestion; BW: body weight; BFR: body fat rate; BMI: body mass index; WBC: white blood cell; RBC: red blood cell; Hb: hemoglobin; Htc: hematocrit; Plt: platelet; AST: aspartate aminotransferase; ALT: alanine aminotransferase; γ-GTP: gamma-glutamyl transpeptidase; ALP: alkaline phosphatase; LDH: lactate dehydrogenase; BUN: blood urea nitrogen; CRE: creatinine; UA: uric acid; TC: total cholesterol; LDL-C: low-density lipoprotein cholesterol; HDL-C: high-density lipoprotein cholesterol; TG: triglyceride; FPG: fasting plasma glucose; HbA1c: hemoglobin A1c.
